# Supplementary material for: Blood pressure variability and early neurological deterioration according to the chronic kidney disease risk categories in minor ischemic stroke patients
Source: PLoS One. 2022 Sep 7;17(9):e0274180. doi: 10.1371/journal.pone.0274180 (PMC9451057; doi:10.1371/journal.pone.0274180)
Supplement: S1 Table — (DOCX) [file pone.0274180.s001.docx]

**S1 Table. Multivariable logistic regression analysis of predictors for END in minor ischemic stroke patients.**

| Variable | aOR (95% CI) | P value | Variable | aOR (95% CI) | P value | Variable | aOR (95% CI) | P value |
| --- | --- | --- | --- | --- | --- | --- | --- | --- |
| Age | 1.02 (0.99-1.05) | 0.137 | Age | 1.01 (0.98-1.04) | 0.397 | Age | 1.02 (0.99-1.04) | 0.287 |
| Adm NIHSS | 1.14 (0.95-1.37) | 0.170 | Adm NIHSS | 1.22 (1.01-1.47) | 0.042 | Adm NIHSS | 1.22 (1.01-1.47) | 0.039 |
| KDIGO classification | | | KDIGO classification | | | KDIGO classification | | |
| Low | Reference |  | Low | Reference |  | Low | Reference |  |
| Moderate | 2.96 (1.49-5.89) | 0.002 | Moderate | 3.15 (1.56-6.38) | 0.001 | Moderate | 3.12 (1.55-6.27) | 0.001 |
| High | 1.00 (0.28-3.52) | >0.999 | High | 1.17 (0.33-4.08) | 0.810 | High | 1.34 (0.39-4.65) | 0.645 |
| Very high | 5.49 (1.73-17.41) | 0.004 | Very high | 6.47 (2.01-20.85) | 0.002 | Very high | 6.6 9(2.12-21.09) | 0.001 |
| SBP mean* | 1.02 (1.00-1.04) | 0.013 | SBP SD* | 1.13 (1.06-1.20) | <0.001 | SBP CoV* | 1.14 (1.04-1.24) | 0.004 |
| Age | 1.03(1.00-1.07) | 0.023 | Age | 1.02 (0.99-1.05) | 0.137 | Age | 1.02 (0.99-1.05) | 0.137 |
| Adm NIHSS | 1.12 (0.93-1.35) | 0.235 | Adm NIHSS | 1.17 (0.98-1.40) | 0.089 | Adm NIHSS | 1.17 (0.98-1.40) | 0.086 |
| KDIGO classification | | | KDIGO classification | | | KDIGO classification | | |
| Low | Reference |  | Low | Reference |  | Low | Reference |  |
| Moderate | 2.93 (1.46-5.85) | 0.002 | Moderate | 3.01 (1.52-5.97) | 0.002 | Moderate | 3.01 (1.52-5.95) | 0.002 |
| High | 1.00 (0.28-3.54) | 0.996 | High | 1.29 (0.37-4.43) | 0.687 | High | 1.28 (0.37-4.41) | 0.694 |
| Very high | 6.59 (2.00-21.71) | 0.002 | Very high | 6.13 (1.99-18.89) | 0.002 | Very high | 6.15 (1.99-18.97) | 0.002 |
| DBP mean* | 1.04 (1.01-1.07) | 0.004 | DBP SD* | 1.00 (0.98-1.03) | 0.832 | DBP CoV* | 1.00 (0.96-1.04) | 0.931 |

* Each BPV parameters were separately adjusted for age, admission NIHSS, KDIGO classification of CKD, which were potentially associated (P < 0.20) with END in univariable logistic regression analysis.

aOR, adjusted odds ratio; CI, confidence interval; adm NIHSS, admission National Institutes of Health Stroke Scale; KDIGO classification, Kidney Disease Improving Global Outcomes classification; SBP, systolic blood pressure; DBP, diastolic blood pressure; SD, standard deviation; CoV, coefficient of variation; BPV, blood pressure variability.
