# Supplementary material for: Blood pressure variability and early neurological deterioration according to the chronic kidney disease risk categories in minor ischemic stroke patients
Source: PLoS One. 2022 Sep 7;17(9):e0274180. doi: 10.1371/journal.pone.0274180 (PMC9451057; doi:10.1371/journal.pone.0274180)
Supplement: S2 Table — (DOCX) [file pone.0274180.s002.docx]

**S2 Table. Logistic regression analysis of SBP and DBP ARV as predictors for END according to renal function.**

| Variables | Normal renal function  (N = 154) | P value | Impaired renal function  (N = 136) | P value |
| --- | --- | --- | --- | --- |
|  | Adjusted OR (95% CI) |  | Adjusted OR (95% CI) |  |
| SBP ARV | 1.36 (1.19−1.56) | <0.001 | 1.35 (1.21−1.51) | <0.001 |
| DBP ARV | 1.05 (0.97−1.15) | 0.210 | 1.60 (1.36−1.90) | <0.001 |

In normal renal function group, age, admission NIHSS, and SBP or DBP mean were adjusted. On the other hand, sex, hyperlipidemia, and SBP or DBP mean were adjusted in impaired renal function group.

OR, odds ratio; CI, confidential interval; NIHSS, National Institutes of Health Stroke Scale; SBP, systolic blood pressure; DBP, diastolic blood pressure.
