# Supplementary material for: Blood pressure variability and early neurological deterioration according to the chronic kidney disease risk categories in minor ischemic stroke patients
Source: PLoS One. 2022 Sep 7;17(9):e0274180. doi: 10.1371/journal.pone.0274180 (PMC9451057; doi:10.1371/journal.pone.0274180)
Supplement: S3 Table — (DOCX) [file pone.0274180.s003.docx]

**S3 Table.** **Interaction between renal function and BPV parameters for the occurrence of END.**

| Interaction | OR (95% CI) for interaction | P for interaction |
| --- | --- | --- |
| Renal function * SBP SD | 1.09 (0.96−1.24) | 0.171 |
| Renal function * SBP CoV | 1.17 (0.98−1.41) | 0.085 |
| Renal function * DBP SD | 1.12 (0.99−1.26) | 0.079 |
| Renal function * DBP CoV | 1.03 (0.93−1.14) | 0.532 |

All models are adjusted for age and admission NIHSS.

BPV, blood pressure variability; END, early neurological deterioration; OR, odds ratio; CI, confidential interval; SBP, systolic blood pressure; SD, standard deviation; CoV, coefficient of variation; DBP, diastolic blood pressure.
