# Supplementary material for: Blood pressure variability and early neurological deterioration according to the chronic kidney disease risk categories in minor ischemic stroke patients
Source: PLoS One. 2022 Sep 7;17(9):e0274180. doi: 10.1371/journal.pone.0274180 (PMC9451057; doi:10.1371/journal.pone.0274180)
Supplement: S4 Table — (PDF) [file pone.0274180.s004.pdf]

Sex	Age	Hypertensio	Diabetes	Hyperlipide	Atrial fibrilla	Heart disea	Smoking	Previous str
0	67	1	0	0	1	0	1	0
1	66	1	1	1	0	0	0	1
1	60	1	0	1	0	0	1	0
1	67	1	0	0	1	0	0	0
1	75	1	1	0	0	0	0	1
1	68	1	1	1	1	0	0	1
0	54	0	0	0	0	0	0	0
1	79	1	0	0	1	0	0	0
1	57	0	0	0	0	0	1	0
1	70	0	1	0	1	1	0	0
1	68	1	1	0	0	1	0	0
1	62	1	1	1	1	0	1	0
1	81	1	0	1	0	1	0	1
1	73	1	0	1	0	0	0	0
1	74	1	0	0	1	1	1	0
0	77	1	1	0	0	0	0	1
0	77	1	1	0	0	0	0	0
0	47	1	0	1	0	0	0	0
1	56	0	0	0	0	0	1	0
0	44	1	0	0	0	1	0	0
1	81	1	1	1	0	0	0	1
1	75	0	0	1	0	0	0	0
0	72	1	0	1	0	0	0	0
1	75	0	0	0	0	0	0	1
0	84	1	0	0	0	0	0	0
1	49	1	1	0	0	0	0	1
1	46	0	0	0	0	0	0	0
1	76	0	0	0	0	0	1	0
1	69	1	0	1	0	0	0	1
1	67	0	0	0	0	0	1	0
1	67	0	0	0	0	0	1	0
0	70	1	0	1	0	0	0	1
1	61	0	0	1	0	0	1	0
1	57	0	0	1	0	0	0	0
0	85	0	0	1	0	0	0	0
0	80	1	1	0	0	0	0	0
1	56	1	0	0	0	0	1	0
0	76	1	0	0	1	0	0	1
0	66	1	1	0	0	0	0	0
1	34	0	0	0	0	0	0	0
1	57	0	0	1	0	0	1	1
1	60	0	0	0	1	0	1	0
1	69	0	0	0	0	0	1	0
1	82	1	0	1	0	0	0	1
1	61	1	0	1	0	0	1	0
0	77	1	1	1	0	1	0	1
1	76	0	0	1	0	1	0	1
1	51	0	0	1	0	0	0	0
0	94	1	0	0	0	0	0	0

0	80	1	1	0	0	0	0	0
1	87	1	0	1	1	0	1	1
1	89	1	1	0	0	0	0	0
0	60	0	0	0	0	0	0	0
1	77	1	0	1	1	0	1	0
0	71	1	1	1	0	0	0	0
0	63	1	0	0	0	1	0	0
1	68	1	1	0	1	0	1	0
1	79	1	0	1	0	1	0	0
1	58	0	0	1	0	1	0	0
1	68	1	0	0	1	0	0	0
1	65	1	1	1	0	0	0	0
0	58	0	0	1	0	0	0	0
0	55	0	0	0	0	0	0	1
0	74	0	1	1	0	0	0	0
1	87	1	1	1	0	0	1	0
1	79	1	1	0	0	0	0	1
0	76	1	0	0	1	0	0	1
0	57	1	0	1	0	0	0	0
0	83	1	1	1	0	0	0	0
0	70	1	1	0	0	0	0	1
1	80	1	0	0	0	0	1	0
0	61	1	0	1	1	0	0	1
1	75	0	0	1	1	1	0	0
1	62	1	0	0	0	0	0	0
0	80	0	0	0	0	0	0	0
0	84	1	1	1	0	0	0	1
1	53	1	0	1	0	0	1	1
1	84	1	0	0	0	0	1	1
0	72	1	1	1	0	1	0	0
0	72	1	0	1	0	1	0	1
1	66	1	0	1	0	1	0	0
0	65	1	1	1	0	0	0	0
1	73	1	1	1	0	0	1	0
1	64	0	1	1	0	1	1	0
0	75	1	1	1	0	0	0	0
1	92	0	0	0	1	1	1	0
1	43	1	0	1	0	0	1	0
1	69	0	0	0	0	0	0	0
1	61	0	0	0	0	0	1	1
1	85	1	0	0	0	0	0	1
1	79	1	1	0	0	0	1	1
1	72	1	0	0	1	0	0	0
0	69	1	0	0	0	0	0	0
0	40	1	1	1	0	0	0	0
0	77	1	0	0	0	0	0	0
1	48	1	0	1	0	0	1	0
0	86	1	0	1	1	1	0	0
0	78	0	1	0	0	0	0	0
0	69	1	1	1	0	1	0	0

1	57	1	0	0	0	0	1	0
0	74	1	0	0	0	0	0	0
1	43	1	0	0	0	0	1	1
1	78	1	0	1	0	0	0	0
1	64	0	0	0	0	0	1	0
0	56	0	0	0	0	0	0	1
1	73	1	1	1	0	0	0	0
1	73	0	0	0	0	0	0	1
0	84	1	1	1	0	0	0	0
1	74	0	0	0	0	0	1	0
1	57	0	0	1	0	0	0	0
1	73	1	1	1	0	0	1	0
1	55	1	1	1	0	0	0	1
0	67	1	1	0	0	0	0	0
0	62	1	0	1	0	0	0	0
1	59	0	0	1	0	0	1	1
1	54	0	0	1	1	0	0	0
1	76	1	0	1	0	0	1	0
0	60	1	0	0	1	0	1	0
1	78	1	0	0	0	0	0	1
1	64	1	0	0	0	0	0	1
1	71	1	0	1	0	0	1	0
1	59	0	0	1	0	0	1	0
0	75	1	0	1	0	0	0	0
1	85	1	1	1	0	0	0	0
1	77	1	0	1	1	0	1	0
1	66	1	0	1	0	1	1	0
1	62	1	0	0	1	0	1	1
0	85	1	0	1	1	0	0	1
1	76	1	0	0	0	0	1	1
1	63	1	0	1	0	0	0	0
0	61	1	0	0	0	0	0	0
1	64	1	0	1	1	0	1	0
0	77	1	1	1	0	0	0	1
0	79	1	1	1	0	0	0	0
1	45	1	0	1	0	0	1	0
1	55	1	0	1	0	0	0	1
1	80	1	0	0	0	1	0	1
0	81	0	1	0	0	0	0	1
1	81	1	1	0	0	0	0	0
0	86	1	0	1	0	0	0	1
1	55	1	1	1	0	0	1	0
0	80	1	0	1	0	0	0	0
1	77	1	0	0	1	0	0	0
0	42	0	0	0	0	1	0	0
1	71	1	0	0	1	1	0	0
0	70	1	0	1	0	0	0	0
1	56	1	1	1	0	0	0	0
1	54	1	0	1	1	0	0	0
0	52	0	1	1	0	0	0	0

1	70	0	0	1	1	0	1	0
1	75	1	1	1	0	0	1	0
0	84	1	0	0	0	0	1	0
0	57	1	1	1	0	0	0	1
1	85	0	0	0	0	0	1	1
1	75	1	0	0	0	0	1	0
1	81	0	0	0	0	0	1	0
1	64	1	0	0	1	0	1	1
1	65	1	0	1	0	0	0	1
1	75	1	0	0	0	0	1	1
1	60	0	0	0	0	0	1	0
0	84	1	1	0	0	0	0	0
0	83	1	0	1	0	0	0	1
0	81	1	0	1	0	0	0	1
0	22	0	0	0	0	0	0	0
0	73	0	0	0	0	0	0	0
1	68	1	0	1	0	0	1	1
0	65	1	0	1	0	0	0	0
0	69	1	0	1	0	0	0	0
1	63	0	0	1	0	1	1	0
1	68	0	0	0	0	0	0	1
0	66	1	1	1	0	0	0	1
1	71	0	0	1	0	0	0	1
0	63	0	0	0	0	0	0	0
1	40	0	0	0	0	0	1	1
1	63	1	1	1	0	1	1	0
1	68	1	1	1	0	1	1	1
1	80	0	0	0	0	0	1	0
0	53	1	0	0	0	0	0	0
1	76	1	0	0	1	0	0	0
0	88	1	0	1	0	0	0	0
1	81	1	0	1	0	0	1	1
1	55	1	1	1	0	0	1	0
1	54	1	0	1	0	0	0	0
1	79	0	1	0	0	0	1	0
0	75	1	1	1	0	0	0	1
0	69	1	1	1	0	0	0	0
0	56	1	0	0	0	0	1	0
1	61	0	1	0	0	1	1	0
0	69	0	1	1	0	1	0	1
0	86	1	1	1	0	0	0	1
1	55	1	0	0	0	0	0	0
1	52	1	1	0	0	0	1	0
0	82	1	1	1	1	1	0	1
0	86	1	1	1	0	0	0	1
0	77	1	1	0	0	0	0	1
1	83	1	1	0	0	1	0	0
0	52	0	0	0	0	0	0	0
1	61	0	1	0	0	1	1	0
1	64	1	0	0	0	0	1	0

1	75	1	1	0	0	1	1	0
0	50	1	0	0	0	0	0	0
1	68	1	0	1	1	0	1	0
0	48	0	0	0	0	0	0	0
1	75	1	1	0	0	0	0	0
0	76	1	0	1	0	1	0	0
1	74	1	0	1	1	0	0	0
1	43	1	0	0	0	0	1	0
1	67	1	0	0	0	1	0	1
1	71	1	0	0	0	1	1	0
1	77	1	0	0	0	0	1	0
1	59	1	0	0	0	0	1	1
0	71	1	0	1	0	0	0	0
1	60	1	1	1	0	0	0	0
1	66	0	0	0	0	0	1	0
1	47	0	0	1	0	0	1	0
0	56	0	0	1	0	0	1	0
1	57	1	0	1	0	0	0	0
1	78	1	1	1	0	1	1	1
1	61	1	1	1	0	0	1	0
0	81	1	0	0	0	0	0	0
1	44	1	1	1	0	0	1	0
0	60	1	0	0	0	0	0	0
1	70	0	0	1	0	0	1	0
1	81	1	0	0	0	1	1	1
1	57	1	0	1	0	0	0	0
1	68	0	1	0	0	0	1	1
1	55	0	1	0	0	0	1	1
1	61	0	0	0	0	0	1	0
1	81	1	1	0	0	0	1	1
1	53	1	0	1	0	0	1	0
1	55	0	0	0	0	0	1	0
1	61	0	0	0	1	1	1	1
1	36	0	0	1	0	0	0	0
0	48	0	0	1	0	0	0	0
1	54	1	0	1	0	0	0	0
1	65	1	0	0	0	0	0	0
1	64	1	0	0	0	0	1	0
1	61	0	1	1	0	0	1	1
0	67	0	0	0	0	0	0	0
1	82	1	1	0	0	1	0	1
1	51	0	0	1	0	0	1	0
1	71	1	0	1	0	0	0	0
0	80	1	0	0	0	0	0	1
1	68	1	0	1	0	0	0	1
0	87	1	1	1	0	0	0	0
1	54	0	1	0	1	0	0	0
0	51	1	1	1	0	0	0	0
1	78	0	0	0	1	1	1	0
1	73	1	0	0	1	0	0	1

1	36	1	0	1	0	1	1	0
0	74	0	0	0	0	0	0	0
1	72	1	0	0	0	0	1	0
0	69	1	0	1	0	1	0	1
1	51	1	0	1	0	0	0	0
1	77	1	1	1	0	0	1	1
1	72	1	0	0	1	0	0	0
1	64	1	0	0	0	0	1	1
1	31	1	0	1	0	0	0	0
1	72	1	0	1	0	0	1	0
1	80	1	0	0	0	0	1	1
1	65	1	1	0	0	0	1	0
0	44	0	0	0	0	0	0	0
1	75	1	0	1	1	0	1	0
0	71	1	0	1	0	0	0	0
1	79	1	0	1	0	0	0	0
0	40	1	0	0	0	0	0	0
1	45	1	0	1	0	1	1	0
0	76	1	0	0	0	0	0	1
0	80	1	0	0	0	0	0	1
0	33	0	0	0	0	0	0	0
1	78	0	1	0	1	0	1	0
0	84	1	0	1	0	1	0	1
1	69	1	0	0	0	0	0	0
1	73	1	0	1	0	0	0	0
1	48	0	0	1	0	0	0	0
1	50	1	1	1	0	0	1	0
1	55	0	0	0	0	0	1	0
0	59	1	0	0	0	0	0	0
0	70	1	0	1	0	0	0	1
0	79	0	0	1	0	0	0	0
0	70	1	0	1	0	0	0	0
1	59	1	0	0	0	0	0	0
1	66	1	1	1	0	0	1	1
1	56	1	1	0	0	0	1	0
1	70	1	0	1	1	1	1	0
1	77	1	1	0	0	0	1	0
0	61	1	1	1	0	0	1	1
0	42	1	0	0	0	0	0	0
0	47	1	0	0	0	0	1	0
0	78	1	1	1	1	0	0	0

eGFR	Albuminuria	NIHSS	KDIGO	END	SBPmean	SBPsd	SBPcov	DBPmean
80	8	1	1	0	157.75	9.761939	6.188234	76
74	211.5	2	2	0	151.6667	10.92398	7.202624	84.16667
63	14.9	2	1	0	141.75	11.01342	7.76961	83.91667
79	9	0	1	0	159.1667	19.82117	12.45309	83.16667
85	34.1	2	2	0	113.9167	9.423938	8.272659	62.41667
64	51.9	4	2	0	145.5833	12.47148	8.566559	72.91667
103	5.1	2	1	0	114.25	8.843333	7.740336	72.16667
69	43.8	0	2	0	171.3333	13.56019	7.914509	81.83333
87	4.8	0	1	0	149.9167	8.867288	5.914811	82.66667
76	31.9	0	2	0	137.25	12.92724	9.418757	57.41667
66	102.9	1	2	0	119.6667	23.36405	19.52427	69.16667
89	5.5	3	1	0	142.1667	11.4084	8.024664	80.75
68	76.4	3	2	1	141.1667	18.06973	12.80028	88.08333
68	72.1	1	2	0	162.6667	18.66613	11.47508	85.75
86	32.6	5	2	0	161.9167	9.976867	6.16173	75.08333
87	92.5	5	2	0	142.4167	23.29049	16.35377	82.33333
68	3375.5	3	3	0	167.75	17.75656	10.58513	82
106	5.3	2	1	0	142.3333	11.48385	8.068278	82.66667
79	11.1	4	1	0	125.5833	10.37881	8.26448	135.0833
114	4.7	3	1	0	141.5	9.375985	6.626138	95.91667
90	21.2	0	1	0	140	19.91345	14.22389	66.41667
77	3.9	0	1	0	110.75	10.03743	9.063142	64.75
90	13	3	1	1	128.75	11.43616	8.882453	67.875
72	47.4	0	2	0	124.1667	10.99449	8.854622	73.91667
94	59.3	3	2	1	132.5	18.15339	13.70067	76.66667
97	9.5	0	1	0	134.5833	18.33258	13.62173	81.75
86	1.9	0	1	0	137.4167	7.786449	5.666306	90.83333
77	4.2	1	1	0	131.9167	10.23771	7.760737	74.58333
90	15.1	1	1	0	131.6667	15.12574	11.4879	70.91667
89	2.3	0	1	0	104.25	11.07926	10.62759	58.25
82	2.3	0	1	0	120.75	11.25833	9.323669	76.33333
78	2.4	1	1	0	133.25	13.11574	9.842959	77
102	204.6	3	2	0	114.8333	10.87811	9.472957	68
92	15.6	5	1	0	133.9167	15.93429	11.89866	76.33333
82	6.4	2	1	0	120.1667	10.18763	8.47792	66.33333
57	67.7	1	3	0	171	15.43314	9.025228	93.08333
82	5.7	1	1	0	142.1667	8.451286	5.944633	100.1667
72	749.7	1	3	0	146.5833	16.18899	11.04422	98.16667
73	22.9	2	1	0	129.3333	20.78607	16.0717	75.33333
115	2.4	0	1	0	127.3333	9.856547	7.740744	77.25
87	19.5	4	1	1	136.6667	11.1243	8.13973	90.44444
84	22.8	1	1	0	126.8333	13.12065	10.3448	75.41667
96	75.5	5	2	0	102.8333	4.302924	4.184367	71.16667
71	24.7	1	1	0	141.75	16.64127	11.73987	70.25
85	9.9	1	1	0	158.1667	24.7086	15.62188	77.08333
60	156.4	0	2	1	147.3333	17.50048	11.87815	69.5
98	9.1	5	1	0	124.25	17.32116	13.94057	78.75
100	4.2	0	1	0	130	9.723449	7.479576	83.25
64	54.3	1	2	0	122.6667	17.71662	14.4429	72.16667

74	26.4	4	1	0	139.5833	9.287904	6.65402	74.16667
55	56.3	2	3	0	162	14.65358	9.045417	85.83333
74	36.3	4	2	1	164.5455	13.86624	8.426999	84.36364
82	9.1	2	1	0	149.0833	15.22234	10.21063	81.5
49	158.6	2	3	0	158.25	11.12021	7.02699	95
64	5407	1	3	0	171.3333	20.32613	11.8635	76.33333
82	8	1	1	0	149.5833	23.16132	15.48389	80.5
84	41.6	4	2	1	136.6667	19.48718	14.25891	93.77778
64	14.2	3	1	1	134.1667	6.462714	4.816929	69.16667
88	17.3	3	1	0	137.3333	17.85972	13.00465	77.16667
90	11.3	1	1	0	167.3333	21.46173	12.82574	97.16667
93	4.1	3	1	0	152.1667	24.08256	15.82644	82.16667
112	66.3	2	2	0	114.8333	8.266398	7.198605	76.16667
87	44.5	4	2	0	130.1667	13.78295	10.58869	81.91667
76	113	4	2	0	116.25	10.09163	8.680968	57
79	50	5	2	0	145.9167	9.709024	6.653814	73.75
57	179.7	3	3	1	134.7143	10.49943	7.793853	76.28571
65	5.9	2	1	0	123.8333	15.59623	12.59453	79.75
70	20.3	2	1	0	153.1667	13.64374	8.907771	90.75
56	17.3	5	2	0	147	19.09307	12.98849	74.58333
95	73.5	5	2	1	162	16.9958	10.49123	66.375
48	1110.2	5	4	0	151.6667	16.96699	11.18703	73.33333
61	269.7	1	2	0	158.1667	12.36442	7.817334	94.91667
78	30.8	1	2	1	123	11.41636	9.281596	75.71429
90	12.8	2	1	0	140.0833	16.64309	11.88085	79.33333
35	137.5	0	4	1	139.6667	16.72958	11.97822	65.33333
52	1082.7	2	4	0	161.5833	14.6378	9.058978	78.75
79	0	5	1	1	158.3333	12.35584	7.803685	94.33333
82	40.6	5	2	1	150.5714	18.57289	12.33494	87.42857
77	2.7	2	1	0	158.3333	10.33382	6.526624	81.16667
54	1473.7	5	4	0	140.9167	11.8587	8.4154	81.91667
74	128.9	2	2	0	130.9167	21.87343	16.7079	69.83333
74	4.4	0	1	0	142.4167	9.567543	6.717994	72.08333
66	10.2	0	1	0	132.3333	12.8228	9.689775	76.83333
73	5.9	3	1	1	130.4444	10.48941	8.041287	75.77778
64	75.9	1	2	1	138.5	12.91124	9.322192	77.16667
40	2254.6	1	4	1	185.1111	15.69589	8.479175	121.8889
115	26.7	0	1	0	141.9167	6.126816	4.317193	86.41667
87	12.4	4	1	0	128.1667	16.14048	12.59335	66.91667
100	11.6	2	1	0	137.3333	6.227992	4.534945	91.08333
56	18.8	4	2	1	121.4167	6.200562	5.106846	75.08333
48	5.4	3	2	1	123.875	9.280356	7.49171	72.25
59	8.6	4	2	0	128.3333	9.575853	7.461704	65.33333
90	52.6	0	2	0	169.5	17.89617	10.55821	80.91667
115	10.2	4	1	0	122.6667	17.38512	14.17265	82.91667
94	6.7	3	1	0	142.1667	12.67663	8.916739	75.16667
77	79.8	2	2	0	153	9.115421	5.957792	95.08333
79	112.7	2	2	0	152.8333	17.52833	11.46892	70.16667
66	7.6	2	1	0	119	11.09463	9.323222	67.16667
47	2.8	1	2	0	146.3333	11.94939	8.165869	87.33333

93	5.1	0	1	0	133.6667	8.467406	6.334718	84.41667
90	11.5	2	1	1	164.2857	22.39579	13.63222	91.57143
111	4.4	1	1	0	132	11.03713	8.36146	90.5
74	22.2	3	1	0	151.9167	9.922045	6.531242	71.66667
63	9.1	1	1	0	115.6667	8.998316	7.779524	73.16667
84	6.1	1	1	0	127.0833	18.53968	14.5886	74
87	9.4	2	1	1	149.8333	8.953584	5.975696	83
86	13.3	0	1	1	120.375	11.81932	9.818753	71.875
77	5.5	5	1	1	147.1667	11.40043	7.746608	75.75
87	13.5	3	1	0	138.75	22.43019	16.16591	74.91667
104	38.8	2	2	1	155.7143	24.52695	15.75125	87.42857
56	16.4	0	2	0	130.0833	6.921092	5.320506	65
79	5.9	2	1	0	132.75	7.747434	5.836108	74.16667
75	88.5	4	2	0	149	10.90454	7.318486	78.41667
89	45.1	5	2	0	157.25	6.397798	4.068552	100.75
83	12.9	5	1	0	122.1667	10.87811	8.904321	75.16667
101	6.1	1	1	0	116.6667	8.742096	7.493225	76.75
76	35.6	2	2	0	153	13.67147	8.935601	73.41667
84	8.9	3	1	0	105.0833	8.016555	7.62876	72.5
62	2.5	2	1	0	128.6667	12.27217	9.537953	83.5
58	3.2	5	2	1	127.25	8.730079	6.860573	80.125
38	723.7	1	4	0	129.9167	6.801849	5.235548	67.66667
67	5	3	1	0	121.3333	10.84882	8.941338	64.5
86	546.2	1	3	0	156.0833	17.9821	11.52084	88.41667
79	508.2	5	3	0	166.5	11.72022	7.039173	79.91667
75	148.9	3	2	1	174.2727	22.18148	12.72803	95.72727
55	55.3	1	3	0	154	8.312094	5.397464	80.41667
77	34.9	5	2	0	136.4167	9.596006	7.034336	85
75	272.9	3	2	0	146.0833	10.72345	7.34064	65.58333
72	5.2	4	1	0	137.9167	8.784887	6.369707	85
87	32.2	2	2	0	125.4167	6.788471	5.412734	82.66667
82	15.2	2	1	0	128.25	17.12056	13.34937	83.58333
78	7.7	1	1	1	131.9	12.2606	9.295372	77.5
46	205.1	5	3	0	138.5833	8.712148	6.286577	87.08333
52	292.9	3	3	0	148.1667	10.76048	7.262415	86.16667
108	5.8	3	1	0	123	8.124038	6.604909	77.41667
97	9.8	2	1	0	125.75	7.460502	5.932805	79.41667
46	25.7	2	2	0	148.25	6.689544	4.51234	77.75
70	19.7	5	1	0	160.5833	12.55865	7.820644	87.33333
85	7.6	2	1	1	141	22.8619	16.21412	79.14286
84	287.6	2	2	0	140.6667	14.77303	10.50215	77.66667
108	11.9	3	1	0	117.25	7.910005	6.746273	72.75
86	126	4	2	1	142.5	15.79701	11.08562	76.08333
77	2461.7	3	3	1	168.2727	16.74569	9.951518	96.45455
108	9	1	1	0	110.9167	3.872005	3.490914	66.83333
69	295.8	4	2	1	114.3333	11.53913	10.09253	75.25
87	10.5	3	1	0	136.4167	8.284249	6.072755	71.5
111	0	0	1	0	156.6667	6.020168	3.842661	83.66667
93	13.1	3	1	1	155.8571	14.69046	9.425591	102.2857
116	30.5	0	2	0	119.9167	9.238834	7.704379	70.75

55	67.9	5	3	0	130.6667	11.89601	9.104092	76.66667
77	4.6	5	1	0	151.5833	10.23771	6.753846	80.5
68	58.2	5	2	0	169.9167	10.68098	6.286011	90.66667
80	15.2	3	1	0	147.4167	7.609305	5.161767	86.16667
68	26.5	4	1	0	157.25	11.67846	7.426682	87.16667
63	5.3	1	1	0	109.3333	14.16355	12.95446	55.5
65	87.8	4	2	0	129.1667	6.685579	5.175932	66.58333
66	4.4	4	1	0	128.5	11.39777	8.869858	77.83333
77	12.2	1	1	0	160.9167	14.56308	9.050076	93.66667
77	192.9	3	2	1	150.6	5.683309	3.773777	85.6
117	13	5	1	0	102.1667	5.356955	5.243349	66.33333
48	88.9	1	3	0	133.8333	10.74357	8.027572	79.91667
71	14.9	5	1	1	160.1667	12.24621	7.645918	79.33333
66	11.5	4	1	0	143.9167	7.890827	5.482914	73.33333
126	3.4	1	1	0	103.25	7.712151	7.469396	57.91667
77	7.2	0	1	0	147.3333	9.178367	6.229661	69.66667
87	4.9	1	1	0	119.0833	8.743396	7.34225	69.08333
91	347.2	5	3	0	163	11.90111	7.301293	83.83333
90	52.2	2	2	0	149.3333	8.003787	5.359679	85
86	10.3	1	1	0	112.5	7.525713	6.689523	65.66667
88	71.7	0	2	1	145.4	21.46936	14.76572	89.4
74	52.3	1	2	0	106.4167	16.42868	15.43807	70.25
57	5	5	2	0	123.9167	6.584532	5.313677	83.75
81	3.3	0	1	0	112.1667	10.76048	9.593294	71.75
108	1.6	2	1	0	112.1667	7.420283	6.615409	71.66667
47	84.5	5	3	0	108.75	6.903556	6.348098	67.83333
88	41.2	3	2	0	127.3333	6.866696	5.392693	76.25
66	11.1	5	1	0	142.75	10.58408	7.414416	74.58333
104	7.5	0	1	0	128.5833	3.82476	2.974538	83.33333
79	5.2	5	1	0	128.6667	13.0477	10.1407	82
81	43.2	0	2	1	141	9.831921	6.972993	80.14286
72	10.3	3	1	0	145.75	11.24217	7.713323	72.83333
64	45.1	2	2	0	146.3333	7.227892	4.939334	87.33333
95	8.9	0	1	0	109.1667	33.33258	30.53366	81.66667
78	19.6	1	1	0	137.1667	7.601834	5.542042	66.41667
18	3369.5	0	4	1	141.75	17.63283	12.43939	87.75
65	26.8	4	1	0	173.9167	18.04771	10.37721	79.58333
98	11.2	4	1	0	114.6667	14.59348	12.72687	72
93	4.5	2	1	0	115.5	5.600325	4.848766	75.75
73	29.4	2	1	0	114.1667	8.621678	7.551835	75.75
54	35.2	4	3	1	147.6	18.83614	12.76161	82
97	18.1	5	1	0	115.1	6.118279	5.31562	71.6
104	200.1	3	2	1	215.6667	4.633213	2.148322	118.3333
81	50.7	3	2	0	114.1667	11.19118	9.80249	65.08333
37	601	1	4	0	145.5	7.982936	5.486554	70.66667
85	57.8	1	2	0	144.9167	13.80684	9.527433	64.91667
28	868.4	4	4	1	168.7778	19.61363	11.62098	93.88889
76	13.4	4	1	0	129.8333	7.986731	6.151526	81.5
82	3.8	0	1	0	113.25	9.573875	8.453753	73.25
82	75	5	2	1	173.1667	13.05815	7.540796	101.1667

25	7.2	0	4	1	110.8889	10.14205	9.146134	57.22222
102	11.6	2	1	0	128.4167	10.38757	8.088954	76.25
69	58.6	1	2	0	161.75	8.81244	5.448185	100.4167
80	21.8	4	1	1	153.6667	8.668997	5.64143	70.66667
78	90.5	4	2	1	172.6	8.203658	4.752988	97.8
83	31.6	4	2	1	143.5833	22.55683	15.70992	75.33333
60	113.9	1	2	0	149.9167	8.151501	5.437355	83
106	13.7	4	1	0	170.9167	4.481443	2.622005	114.5833
55	31.9	2	3	0	126.75	7.794229	6.149293	76.75
62	54.4	0	2	1	118.8571	11.11198	9.349025	77.42857
90	4.2	3	1	0	140.3333	10.78158	7.682835	72.25
90	22.9	1	1	0	142.4167	7.971065	5.597003	83.66667
91	22	2	1	0	172.25	10.07359	5.84824	94.91667
93	24.5	1	1	0	164.1667	7.469128	4.549723	92.08333
81	50.3	1	2	0	114.4167	7.704288	6.733537	72.91667
86	2.1	0	1	0	112.5833	3.203928	2.845828	72.16667
84	39.6	1	2	0	124.25	8.475902	6.821651	78.41667
77	44.4	5	2	0	169.4167	6.881574	4.061923	102.5833
16	1264.7	2	4	0	145.5833	8.826183	6.062633	80.33333
44	9070	5	4	0	167.25	7.123903	4.259434	88.33333
66	22.5	2	1	0	161	7.135061	4.431715	79.75
87	25.1	5	1	0	172.1667	8.663752	5.032189	115.25
107	7.9	0	1	0	153.9167	7.668807	4.982441	83.08333
92	42.7	3	2	1	130.5	11.82832	9.063845	79.66667
41	2421.7	0	4	0	110.4167	6.815201	6.172258	55.33333
94	19.9	2	1	0	178.4167	9.009675	5.049794	91.83333
101	8.1	0	1	0	106.1667	5.131601	4.833534	70.75
86	65.5	5	2	0	125.5833	6.721179	5.351967	78.5
82	62.9	4	2	1	133.75	10.68878	7.991611	83.5
58	26.2	4	2	0	117.25	12.2558	10.4527	66.75
83	17.9	0	1	0	135.5	4.852366	3.581082	82.58333
106	8.6	4	1	0	131	13.38928	10.22082	78.25
68	35.8	2	2	1	104.1667	10.0681	9.665377	66.5
105	1.4	1	1	0	140.9167	7.948223	5.640371	89.08333
98	104.8	0	2	0	138.0833	6.33114	4.585014	85.41667
107	101.5	5	2	1	127.0833	14.52558	11.42996	78.41667
91	7.8	3	1	0	145.25	10.85546	7.473635	76.91667
98	11.3	4	1	0	196.1667	7.732262	3.94168	99.5
72	34.2	1	2	1	155.5714	11.68842	7.513214	77.85714
84	7	0	1	0	133.6667	13.11026	9.808171	68.91667
56	2.9	2	2	0	139.8333	12.52513	8.957182	81.41667
83	2.4	1	1	0	106.3333	7.126561	6.702095	66.41667
87	47.7	4	2	0	130.0833	7.982462	6.136422	77.25
86	19.3	5	1	1	140.4286	10.72158	7.634903	70.85714
64	3.8	0	1	0	148.8333	11.20741	7.530175	78.66667
80	30.1	1	2	0	141	14.77713	10.48024	73.08333
83	42.9	2	2	0	146.4167	8.607168	5.878544	87
56	3.5	4	2	0	124.1667	9.580599	7.715919	81.08333
85	4.4	3	1	0	149.6667	8.689945	5.8062	77.58333
82	3.8	2	1	0	146.0833	6.542981	4.478938	80.5

91	155.3	5	2	0	160.5	5.664884	3.529523	99.25
52	7.4	0	2	0	127.8333	7.259268	5.678697	72.5
19	167.6	1	4	1	116.3333	11.18332	9.61317	52
80	10.2	2	1	0	140.5833	10.01325	7.122643	80.33333
100	101.2	0	2	0	150.75	10.72063	7.111526	88.75
45	251.7	5	3	0	157.5833	7.50101	4.760028	70.66667
65	168.9	3	2	0	155	8.675986	5.59741	76.66667
88	4.3	2	1	0	136.8333	5.702206	4.167264	64.83333
106	60.5	5	2	0	150.0833	15.72756	10.47922	99.25
91	5.6	0	1	0	137.2222	18.1506	13.22716	78
49	282.3	1	3	0	123.0833	14.49425	11.77597	66.83333
96	23	0	1	0	162.0833	8.106769	5.001605	83.58333
100	0	0	1	0	112.75	7.581377	6.724059	81.41667
62	6.2	4	1	0	107.6667	5.399214	5.01475	70.91667
74	48.6	0	2	0	140.0833	9.199391	6.567084	78.91667
66	28.5	2	1	0	147.5	15.72202	10.65899	79.91667
100	10.7	2	1	0	117.1667	5.605733	4.784409	77.33333
98	5.2	5	1	0	149.5833	6.639528	4.438682	94.16667
65	17.2	5	1	0	141.5	10.97518	7.75631	78.5
66	9.5	3	1	0	125.25	11.28253	9.008007	63.16667
115	10.4	2	1	0	120.4167	6.052172	5.026025	76.75
89	4.8	2	1	0	122.6667	9.893371	8.065248	74.25
60	9.8	3	1	0	136.3333	12.68022	9.300892	74.83333
28	114.6	3	4	1	163.4286	20.91138	12.79542	85.42857
78	4	5	1	0	142	5.308655	3.738489	79.16667
91	12.4	1	1	0	132.5	9.968131	7.523118	85.75
45	6691.4	0	4	1	164.4444	14.18724	8.627376	79.66667
108	4.1	0	1	0	127.9167	5.418123	4.235666	78.91667
94	6.3	0	1	1	126.2222	13.44226	10.64968	77.33333
81	6.8	0	1	0	139.6667	8.094143	5.795329	76.91667
85	298	2	2	0	134.8333	9.163498	6.796167	87.41667
94	23	1	1	0	145	10.01817	6.90908	78.83333
67	4.9	1	1	0	139.9167	9.080031	6.4896	89.75
91	76.3	4	2	0	124.5	3.233349	2.597067	60.58333
95	69.9	3	2	1	160.4545	10.32825	6.436869	97.63636
40	47.2	2	4	0	104.5833	9.189503	8.786776	32.41667
88	56.1	3	2	0	113.0833	4.294994	3.798078	60.08333
105	3257.5	4	3	1	179.1111	20.07763	11.20959	91.44444
89	11.9	3	1	1	182.3333	12.20656	6.694637	105
91	15	4	1	0	143.25	10.66963	7.448255	80.25
56	16.7	3	2	0	113.0833	8.918401	7.886574	60.91667

DBPsd	DBPcov
10.31327	13.5701
7.952511	9.448527
8.554194	10.19368
3.537676	4.253719
9.8577	15.79338
10.14852	13.91797
9.561603	13.24933
8.009464	9.787533
7.487363	9.057294
7.329372	12.76523
17.39296	25.14645
8.091803	10.02081
8.753787	9.938074
8.540013	9.959199
6.721179	8.951625
8.731691	10.60529
4.8053	5.860122
5.122736	6.196858
219.0813	162.1824
7.012435	7.310966
5.177896	7.796079
5.738625	8.862741
5.938675	8.749429
5.976596	8.085586
16.1433	21.05648
9.036542	11.05387
5.621927	6.189277
4.581749	6.143127
8.732992	12.31444
7.325237	12.57551
5.597619	7.333125
3.861229	5.014583
7.36083	10.82475
6.227992	8.158941
6.800178	10.25153
9.709024	10.43046
6.617241	6.606231
10.24103	10.43229
6.372288	8.45879
6.210329	8.03926
5.052502	5.586305
11.08199	14.69436
3.157483	4.436744
10.3934	14.79487
9.894519	12.83613
10.36822	14.9183
6.52443	8.28499
8.82275	10.5979
10.79422	14.95735

6.206058	8.367718
5.78137	6.735577
9.113427	10.80255
6.403124	7.856594
11.69304	12.30847
6.733003	8.820528
6.598898	8.197389
13.12229	13.99296
7.960318	11.50889
6.860073	8.889944
10.79422	11.10897
6.249848	7.606306
6.249848	8.20549
8.151501	9.950968
8.591751	15.07325
5.8329	7.909018
4.75094	6.227824
12.97638	16.27132
10.20806	11.24855
7.774765	10.42427
7.744814	11.66827
4.92366	6.714081
9.558036	10.06992
6.600866	8.718125
9.267081	11.68119
9.257266	14.16928
13.37654	16.98608
8.066391	8.550945
14.10505	16.13322
5.356955	6.599945
7.821396	9.547992
12.02145	17.21448
5.930788	8.227682
7.814129	10.17023
3.700601	4.88349
7.547627	9.780942
16.33588	13.40228
5.517877	6.3852
6.097068	9.111435
3.315483	3.640054
5.58339	7.436257
6.341473	8.777126
6.91945	10.59099
13.41951	16.58436
12.42035	14.97932
8.177945	10.87975
5.712161	6.007532
11.31237	16.12214
15.60206	23.22887
9.442008	10.81146

6.402533	7.584441
9.180725	10.02575
8.073526	8.921023
5.757735	8.034049
4.951278	6.767122
5.799687	7.837414
9.208692	11.09481
7.989949	11.11645
9.668365	12.76352
11.88168	15.85986
14.89807	17.04027
4.729021	7.275416
7.158381	9.65175
10.50072	13.39093
10.91392	10.83267
7.420283	9.871774
8.158933	10.63053
4.718596	6.427146
6.653775	9.177621
6.895321	8.25787
5.24915	6.551201
9.276886	13.70968
9.995454	15.49683
9.680893	10.94917
4.440687	5.556646
10.61217	11.08584
4.776045	5.939123
11.32977	13.32914
7.727852	11.78326
3.045115	3.582489
5.613836	6.79093
10.88333	13.02094
9.947082	12.83494
5.350588	6.144215
8.912028	10.34278
4.981025	6.434047
6.515134	8.203736
7.162085	9.211684
8.060378	9.229441
8.513295	10.75687
2.994945	3.856153
4.864061	6.685995
17.0585	22.42081
7.407613	7.6799
5.254147	7.861567
6.877169	9.139095
4.776838	6.680892
4.735424	5.65987
16.81977	16.44391
4.938255	6.979866

9.575853	12.49024
6.667424	8.282515
6.300553	6.949139
5.702206	6.617647
8.891603	10.20069
6.459665	11.63904
7.573378	11.37429
7.432525	9.549282
9.884178	10.5525
5.319774	6.21469
4.417596	6.659692
4.501683	5.632972
6.971805	8.787989
12.08555	16.4803
11.14743	19.24736
5.72607	8.219239
7.115391	10.29972
7.246734	8.644215
5.575922	6.559909
2.994945	4.56083
6.131884	6.858931
5.594234	7.963322
4.330127	5.170301
6.52443	9.093282
3.498918	4.882211
6.191392	9.127359
7.123903	9.342824
11.35748	15.22791
4.334499	5.201398
11.95446	14.57861
5.241774	6.540537
11.08507	15.21978
7.535773	8.628748
6.958753	8.520922
8.607168	12.95935
9.032349	10.29328
8.4257	10.58727
6.835735	9.494076
3.519427	4.646108
5.395705	7.123043
11.11306	13.55251
5.815688	8.122469
8.869423	7.495287
10.84149	16.65786
17.46338	24.71233
16.46185	25.35843
6.936217	7.387687
3.605551	4.423989
5.119038	6.988447
8.922223	8.819331

4.711098	8.232987
8.335303	10.93154
16.22265	16.15533
3.284491	4.647864
8.136338	8.319364
11.32442	15.03241
6.564367	7.908875
7.948223	6.936631
5.626641	7.331129
7.870983	10.16548
5.06548	7.011045
8.804269	10.52303
9.238834	9.733627
6.680478	7.254818
4.209477	5.772997
3.433348	4.757526
9.8577	12.57092
7.140898	6.96107
9.403417	11.7055
6.429101	7.278227
4.454314	5.585346
10.2879	8.926595
7.089792	8.533351
6.945677	8.718423
5.710172	10.31959
8.332121	9.07309
4.15878	5.878135
8.949352	11.40045
11.56143	13.84602
6.017021	9.014264
7.867752	9.527045
6.743684	8.618126
7.867655	11.83106
7.948223	8.922233
3.776924	4.421764
9.443211	12.04235
7.879298	10.24394
5.551413	5.579309
10.15593	13.04431
9.307459	13.50538
6.666856	8.188564
5.451577	8.208147
7.545318	9.767401
5.273474	7.442402
6.541244	8.315141
11.05736	15.12979
7.781096	8.943788
6.229816	7.683226
4.907477	6.325427
8.129632	10.09892

7.225271	7.27987
5.807519	8.010371
6.292853	12.10164
6.065301	7.550167
8.677295	9.777234
9.698485	13.72427
5.597619	7.301242
6.846809	10.56063
5.925676	5.970454
7.158911	9.17809
10.95307	16.38863
5.869154	7.021919
6.907944	8.484681
5.775471	8.144024
6.721179	8.516805
5.976596	7.478535
3.312053	4.282828
7.17107	7.615295
9.91326	12.62836
6.322159	10.0087
4.673426	6.089154
7.735691	10.41844
4.895886	6.542386
11.57378	13.5479
6.820202	8.614992
5.189587	6.051997
3.535534	4.437909
4.399552	5.574934
8.774964	11.34694
7.609305	9.89292
6.359793	7.275264
7.68509	9.748528
7.436091	8.285339
7.798116	12.87172
5.390227	5.520717
6.707639	20.69195
3.260182	5.426101
14.22244	15.55309
10.95445	10.43281
5.327885	6.63911
7.316958	12.01142
