# Supplementary figures and images for: Blood pressure variability and early neurological deterioration according to the chronic kidney disease risk categories in minor ischemic stroke patients
Source: PLoS One. 2022 Sep 7;17(9):e0274180. doi: 10.1371/journal.pone.0274180 (PMC9451057; doi:10.1371/journal.pone.0274180)

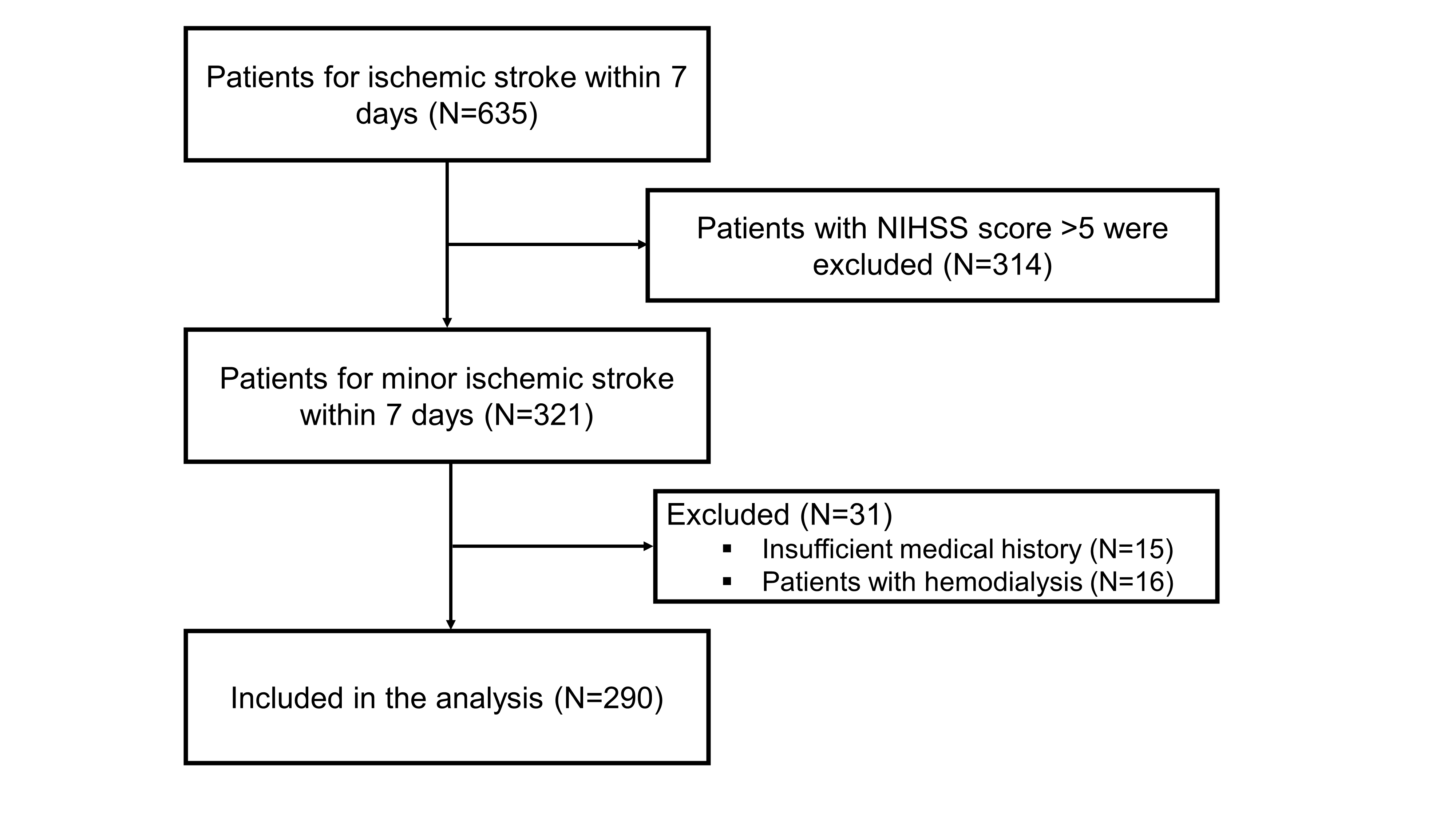

Supplement: S1 Fig — NIHSS, National Institutes of Health Stroke Scale. (TIF) [file pone.0274180.s005.tif]
