# Supplementary material for: Blood pressure variability and early neurological deterioration according to the chronic kidney disease risk categories in minor ischemic stroke patients
Source: PLoS One. 2022 Sep 7;17(9):e0274180. doi: 10.1371/journal.pone.0274180 (PMC9451057; doi:10.1371/journal.pone.0274180)
Supplement: Attachment — Submitted filename: CKD and END Response to Reviewers [PLOS ONE].docx [file pone.0274180.s006.docx]

**Response to the Reviewers’ Comments**

Manuscript Number: PONE-D-22-18870

My co-authors and I are pleased to submit the revised manuscript entitled, ‘Blood Pressure Variability and Early Neurological Deterioration according to Renal Function in Minor Ischemic Stroke Patients’. We have carefully responded to the reviewer’s comments below and have updated the manuscript accordingly. The responses are presented as blue letters and the changes are presented as red letters in the manuscript. We hope that the Editor and the reviewers will ultimately consider our manuscript acceptable for publication in *PLOS ONE.*

**Review Comments to the Author**

**Reviewer #1**

Jae-Chan Ryu and coworkers evaluated the relationship of inpatient blood pressure variability and CKD risk category with early neurological deterioration in patients with mild stroke. They report intriguing findings, but I have several reservations and suggestions.

Major comments:

**Comment 1:** The Title and several additional phrases are misleading in that not “Renal Function” is studied but rather CKD risk categories. The authors must choose whether studying relationships with kidney function (e.g. eGFR, serum creatinine) or CKD risk categories.

**Response 1:** We appreciate the reviewer’s instructive comment. We agree that CKD risk categories were studied rather than renal function in this research. Therefore, we changed the title as below.

“Blood Pressure Variability and Early Neurological Deterioration according to the Chronic Kidney Disease Risk Categories in Minor Ischemic Stroke Patients.”

**Comment 2:** In my opinion, BP variability should not by analyzed outside the context of blood pressure. the SD of BP, and to a lesser extent the CoV of BP, are both dependent on the blood pressure mean. Thus, SBP (or DBP) should be included in all models, alongside the BP variability index (SD or CoV), and the authors should consider also average real variability [ARV] and variability independent of the mean [VIM]).

**Response 2:** We greatly appreciate the careful comment of reviewer. The reviewer’s comment is reasonable because SD and CoV are dependent on the BP mean. From the previous studies, BPV predicted the risk of cardiovascular disease independent to the mean BP. Therefore, we added SBP or DBP mean in all models (multivariable analysis in Table 3).

**Table 3.**

| Variables | Normal renal function  (N = 154) | P value | Impaired renal function  (N = 136) | P value |
| --- | --- | --- | --- | --- |
|  | Adjusted OR (95% CI) |  | Adjusted OR (95% CI) |  |
| SBP mean, mmHg | 1.02 (0.99−1.06) | 0.106 | 1.02 (1.00−1.04) | 0.062 |
| SBP SD | 1.06 (0.96−1.19) | 0.254 | 1.15 (1.06−1.26) | 0.001 |
| SBP CoV | 1.08 (0.93−1.25) | 0.298 | 1.23 (1.09−1.40) | 0.001 |
| DBP mean, mmHg | 1.02 (0.98−1.07) | 0.338 | 1.04 (1.00−1.07) | 0.027 |
| DBP SD | 0.99 (0.94−1.03) | 0.527 | 1.08 (0.96−1.22) | 0.188 |
| DBP CoV | 0.98 (0.93−1.04) | 0.517 | 1.06 (0.96−1.16) | 0.238 |

SBP and DBP mean were adjusted for age and admission NIHSS, which were potentially associated (P < 0.20) with END in univariable logistic regression analysis. Moreover, SBP or DBP SD, and SBP or DBP CoV were additionally adjusted for SBP or DBP mean.

After adjusting mean BP, still BPV parameters were independently associated with END only in the impaired renal function group.

Moreover, another reviewer also mentioned about the adjustment for additional confounding factors, and another reviewer recommended to separately perform the univariable analysis in each group. Therefore, we separately analyzed the univariable analysis in normal renal function group and impaired renal function group. Age and admission NIHSS were the potential factors (P<0.20) in normal renal function group. On the other hand, sex and hyperlipidemia were the potential factors in impaired renal function group. Therefore, SBP and DBP mean were adjusted for age and admission NIHSS in normal renal function group, and adjusted for sex and hyperlipidemia in impaired renal function group. The results are as below.

**Table 3.**

| Variables | Normal renal function  (N = 154) | P value | Impaired renal function  (N = 136) | P value |
| --- | --- | --- | --- | --- |
|  | Adjusted OR (95% CI) |  | Adjusted OR (95% CI) |  |
| SBP mean, mmHg | 1.02 (0.99−1.06) | 0.106 | 1.02 (1.00−1.04) | 0.062 |
| SBP SD* | 1.06 (0.96−1.19) | 0.254 | 1.20 (1.09−1.32) | <0.001 |
| SBP CoV* | 1.08 (0.93−1.25) | 0.298 | 1.30 (1.12−1.50) | <0.001 |
| DBP mean, mmHg | 1.02 (0.98−1.07) | 0.338 | 1.04 (1.00−1.07) | 0.027 |
| DBP SD* | 0.99 (0.94−1.03) | 0.527 | 1.13 (0.99−1.28) | 0.064 |
| DBP CoV* | 0.98 (0.93−1.04) | 0.517 | 1.09 (0.99−1.20) | 0.091 |

SBP and DBP mean were adjusted for were adjusted for potential factors (P <0.20) for END. In normal renal function group, age and admission NIHSS were adjusted. On the other hand, sex, and hyperlipidemia were adjusted in impaired renal function group.

*SBP SD and CoV were additionally adjusted for SBP mean, and DBP SD and CoV were additionally adjusted for DBP mean.

This result also showed no statistically significant differences with minor change of odds ratio and P value.

Moreover, ARV and VIM are recently emerging parameters for BPV. However, in case of variability independent of the mean (VIM), this method is mainly used in long-term BPV such as visit-to-visit variability for the long-term management of hypertension (*EClinicalMedicine*. 2022;48: 101442, *Am J Hypertens.* 2017;30(2):112–114). The comparison of various evaluation of BPV is described in the below Table (H.Y Lee, Blood Pressure Variability and Cardiovascular Risk, *J Korean Diabetes* 2014;15:206-210).

As the reviewer’s comment, we added SBP and DBP ARV in S2 Table. SBP and DBP ARV in normal renal function group were adjusted for age, admission NIHSS, and SBP or DBP mean. Also, SBP and DBP ARV in impaired renal function group were adjusted for sex, hyperlipidemia, and SBP or DBP mean. Since the results of ARV showed the similar trend with SD and CoV, we only added the result to the S2 Table to make our main manuscript more concise.

**S2 Table. Logistic regression analysis of SBP and DBP ARV as predictors for END according to renal function**

| Variables | Normal renal function  (N = 154) | P value | Impaired renal function  (N = 136) | P value |
| --- | --- | --- | --- | --- |
|  | Adjusted OR (95% CI) |  | Adjusted OR (95% CI) |  |
| SBP ARV | 1.36 (1.19−1.56) | <0.001 | 1.35 (1.21−1.51) | <0.001 |
| DBP ARV | 1.05 (0.97−1.15) | 0.210 | 1.60 (1.36−1.90) | <0.001 |

In normal renal function group, age, admission NIHSS, and SBP or DBP mean were adjusted. On the other hand, sex, hyperlipidemia, and SBP or DBP mean were adjusted in impaired renal function group.

**Comment 3:** When hypothesizing that the association between END and BPV is conditional upon CKD risk category, an appropriate interaction term must be included and tested in the statistical models (e.g. END ~ SD(SBP)*[CKD risk category]). It is not sufficient to claim that the association was only significant in a certain group and not the other.

**Response 3:** Thank you for the reviewer’s comment. According to the reviewer’s suggestion, we tested in the statistical models including interaction term. The interactions between renal function (normal vs. impaired) and all BPV parameters were analyzed.

**S3 Table.**

| Interaction | OR (95% CI) for interaction | P for interaction |
| --- | --- | --- |
| Renal function * SBP SD | 1.09 (0.96−1.24) | 0.171 |
| Renal function * SBP CoV | 1.17 (0.98−1.41) | 0.085 |
| Renal function * DBP SD | 1.12 (0.99−1.26) | 0.079 |
| Renal function * DBP CoV | 1.03 (0.93−1.14) | 0.532 |

All models are adjusted for age and admission NIHSS.

When we tested the interaction between renal function and BPV parameters, SBP CoV and DBP SD approached the borderline of significance in our study. Especially, the odds ratios of both parameters were more than 1.00, therefore, there might be some synergic interaction between renal function and some BPV parameters for END. Maybe small sample size may limit the statistical significance, and further verification in a large sample size with prospective nature could be helpful. As the reviewer’s mentioned, Interaction term in this study is quite important, so we added this table as S3 Table.

**Comment 4:** Information about “medications stopped” should be presented and included in the models, as this clinical management decision (please cite an appropriate justification) surely increases BP variability.

**Response 4:** In our stroke center, the center protocol recommends discontinuing the blood pressure lowering medication during the acute stage of ischemic stroke, mostly during the admission period (*Stroke*. 2019;50:e344-e418). In patients with acute ischemic stroke, early treatment of hypertension is indicated when required by comorbid conditions such as concomitant acute coronary event, acute heart failure, aortic dissection, post-fibrinolysis sICH, or preeclampsia/eclampsia. There was no patient who indicated early treatment for hypertension. All patients included in this study discontinued any type of anti-hypertensive medication. Moreover, we have obtained the type of anti-hypertensive medication which was used before admission and were presented as below. There was no significant difference according to the groups.

| Characteristic | The risk of KDIGO classification | | | | P value |
| --- | --- | --- | --- | --- | --- |
|  | Low  (N = 154) | Moderate  (N = 97) | High  (N = 22) | Very high  (N = 17) |  |
| BP lowering agent  at admission | 72 (46.8) | 55 (56.7) | 18 (81.8) | 14 (82.4) |  |
| RAS inhibitors | 54 (75.0) | 37 (67.3) | 10 (55.6) | 7 (50.0) | 0.172 |
| β-blockers | 9 (12.5) | 12 (21.8) | 3 (16.7) | 5 (35.7) | 0.175 |
| CCBs | 43 (59.7) | 38 (69.1) | 12 (66.7) | 8 (57.1) | 0.681 |
| Diuretics | 17 (23.6) | 8 (14.5) | 2 (11.1) | 2 (14.3) | 0.448 |
| Antithrombotic agent at admission | 54 (35.1) | 47 (48.5) | 13 (59.1) | 12 (70.6) | 0.801 |
| Antiplatelet agent | 39 (72.2) | 39 (83.0) | 9 (69.2) | 10 (83.4) |  |
| Anticoagulation | 12 (22.2) | 6 (12.8) | 3 (23.1) | 1 (8.3) |  |
| Both | 3 (5.6) | 2 (4.2) | 1 (7.7) | 1 (8.3) |  |

RAS, renin-angiotensin system; CCBs, calcium channel blockers.

Minor comments:

**Comment 5:** Line 29 “estimated glomerulus filtration ratio (eGFR)” - glomerulus should be glomerular and ratio should be rate.

**Response 5:** We greatly appreciate the careful comments of reviewer regarding spelling errors in manuscript. We corrected as below. We apologize for the spelling error which were the reviewer’s concerns.

“The severity of CKD is defined by the estimated glomerular filtration rate (eGFR), which represents the residual renal function, and the severity of proteinuria, which is the result of increased permeability of the damaged capillary wall and impaired resorption.”

**Comment 6:** Lines 133-134 please also present ranges and p-value.

**Response 6:** We added the standard deviation and p-value. Since we excluded BP data after END due to additional factor such as induced hypertension treatment, there was difference in the number of measurements between the two groups.

“In patients without END, the BP was measured 12.0 ± 0.2 times on average, and in those with END, the BP was measured 9.5 ± 1.6 times on average (*P* < 0.001).”

**Comment 7:** Line 143 - neurological severity on admission was LOWER in the highest CKD risk group according to the respective table.

**Response 7:** Thank you for the reviewer’s careful comment. Because admission NIHSS was lower in the very high risk group, we corrected the sentence.

“The prevalence of hypertension, diabetes, and history of coronary artery disease also increased (*P* = 0.002, *P* < 0.001, and *P* = 0.008, respectively). There were also significant differences in neurological severity at admission and on discharge. (*P* = 0.025 and *P* < 0.001, respectively).”

**Comment 8:** Figure 1 is intriguing, but some values may be concealed due to the semi-3D nature. please provide all values in a supplementary table.

**Response 8:** We added all values and changed Figure 1 as 3D graph and table with all detailed value.

**Comment 9:** Table 2 - albuminuria should be presented as median and 25th-75th percentile.

**Response 9:** Thank you for the reviewer’s comment. We presented albuminuria as median and 25th-75th percentile.

| Albuminuria, mg/g creatinine | 15.2 [6.3−52.0] | 41.6 [13.8−120.3] | <0.001 |
| --- | --- | --- | --- |

**Reviewer #2**

**Comment 1:** Interesting study evaluating the effects of BPV on END in patients with and without CKD. In patients with minor stroke, study does show age as well as BP along with CKD affect neurological outcomes. Advanced CKD patients usually have atherosclerotic diseases that might be confounding factor. Nevertheless an interesting article to prove CKD is an important risk factor for stroke outcomes. Would consider authors to comment on whether the effect of CKD on END can be independent of BPV.

**Response 1:** We greatly appreciate the reviewer’s positive comment. Because of multi-directional association among CKD, BPV, and END, it would be quite difficult to prove the causality and independency among these variables. In our additional analysis, there were quasi-significant interactions between some BPV parameters and renal function, but these interactions do not guarantee the independence between renal function and BPV (We added this table as S3 Table because of the importance of interaction term).

**S3 Table.**

| Interaction | OR (95% CI) for interaction | P for interaction |
| --- | --- | --- |
| Renal function * SBP SD | 1.09 (0.96−1.24) | 0.171 |
| Renal function * SBP CoV | 1.17 (0.98−1.41) | 0.085 |
| Renal function * DBP SD | 1.12 (0.99−1.26) | 0.079 |
| Renal function * DBP CoV | 1.03 (0.93−1.14) | 0.532 |

All models are adjusted for age and admission NIHSS.

However, previous studies showed the association between CKD and END, and other studies also suggested the association between BPV and END. Since BPV is associated with CKD, there might be some direct pathway from CKD to END, and there might exist indirect pathway from CKD to END via BPV. Further prospective investigation with large sample size would be helpful to solve this complicated question.

**Reviewer #3**

The authors revealed that the association between END and BPM parameters differs according to renal function in minor ischemic stroke; BPV was associated with END in patients with renal impairment, but less in those with normal renal function. These findings are interesting, and the manuscript is well written, hence the article would have potential for acceptation. Although several points should be described clearly.

**Comment 1:** I basically agree with your data and conclusion, but there were some discrepancies in patients with high risk levels of CKD patients. These data were seen to be severe outcomes in patients with moderate risk than high risk. Could the authors explain this inverted phenomenon?

**Response 1:** We appreciate the reviewer’s instructive and pertinent comment. As the reviewer’s mentioned, there were some discrepancies in patients with high risk group of CKD patients (30.9% in moderate risk group vs. 18.2% in high risk group). It seems to be that the number of high, and very high risk group was small compared with low or moderate risk group. Small sample size in high and very high risk group was a limitation of this study. But in this pilot study, we showed the effects of BPV on END are associated with renal function in minor ischemic stroke patients. Due to small sample size in high and very high risk group, we divided patients into normal renal function vs. impaired renal function group. Moreover, BPV was associated with END in patients with impaired renal function, but less in those with normal renal function. Although we showed the effect of CKD and BPV on END, there were some discrepancies in patients with high risk group of CKD patients, and it may be caused by small number of high and very high risk group. Further research with large study population must be needed. Therefore, we emphasized the first limitation as below.

“First, because we performed the study at a single center with a small sample size, it is difficult to generalize the results. Especially, the number of high risk and very high risk group was too small. Therefore, the findings require further verification in a larger, prospective study.”

**Comment 2:** When the authors confirmed the creatinine levels and determined the CKD stage? The creatinine levels may increase at the timing of admission (due to dehydration or some reason). Could the authors describe the timing for measurement the creatinine levels? Moreover, if the authors determined the CKD stage at admission, could the authors show the change of creatinine levels?

**Response 2:** Thank you for the reviewer’s careful comment. Creatinine levels were measured on the first admission day in the emergency department, before the treatment for stroke. We added some sentences as below. Moreover, as the reviewer’s commented, creatinine levels may increase at the timing of admission. However, our center did not test creatinine regularly in clinical practice. Consequently, we could not obtain follow-up data for creatinine in this retrospective study. According to this limitation, we added a sentence about the method and timing of creatinine measurement, and corrected some sentences in limitation section.

“Creatinine level and eGFR and were measured on the first admission day in the emergency department. Moreover, albuminuria was estimated as the urine albumin/creatinine ratio obtained from spot urine analysis on the first admission day”

“Second, we measured albuminuria and creatinine only once on the day of admission. These parameters can fluctuate according to the sampling time or stress of the stroke. Follow-up data for albuminuria and creatinine may have strengthened our results.”

**Comment 3:** Could the authors show the medication for antihypertensive agents and antithrombotic agents at the time of admission? The class effect of antihypertensive drug on prevention for stroke was observed in the recent report (Zhu et al. Cochrane Database of Systematic Reviews 2022, Issue 1. Art. No.: CD003654.).

**Response 3:** We appreciate the reviewer’s instructive comment. As the reviewer’s mentioned, there was some different effects for stroke or cardiovascular events according to the class of anti-hypertensive medication in recent study. Therefore, we additionally investigated the type of anti-hypertensive medication and antithrombotic agents before stroke. In our study, there were no differences in the class of anti-hypertensive drugs or antithrombotic agents. Additional data about medication are as below (Statistical analysis in BP lowering agent was performed separately, because there were so many combinations in description of anti-hypertensive agent). This result was added to Table 1.

| Characteristic | The risk of KDIGO classification | | | | P value |
| --- | --- | --- | --- | --- | --- |
|  | Low  (N = 154) | Moderate  (N = 97) | High  (N = 22) | Very high  (N = 17) |  |
| BP lowering agent  at admission | 72 (46.8) | 55 (56.7) | 18 (81.8) | 14 (82.4) |  |
| RAS inhibitors | 54 (75.0) | 37 (67.3) | 10 (55.6) | 7 (50.0) | 0.172 |
| β-blockers | 9 (12.5) | 12 (21.8) | 3 (16.7) | 5 (35.7) | 0.175 |
| CCBs | 43 (59.7) | 38 (69.1) | 12 (66.7) | 8 (57.1) | 0.681 |
| Diuretics | 17 (23.6) | 8 (14.5) | 2 (11.1) | 2 (14.3) | 0.448 |
| Antithrombotic agent at admission | 54 (35.1) | 47 (48.5) | 13 (59.1) | 12 (70.6) | 0.801 |
| Antiplatelet agent | 39 (72.2) | 39 (83.0) | 9 (69.2) | 10 (83.4) |  |
| Anticoagulation | 12 (22.2) | 6 (12.8) | 3 (23.1) | 1 (8.3) |  |
| Both | 3 (5.6) | 2 (4.2) | 1 (7.7) | 1 (8.3) |  |

RAS, renin-angiotensin system; CCBs, calcium channel blockers.

“There were no differences in the use of anti-hypertensive medication and antithrombotic agent before admission according to the risk of KDIGO classification.”

**Comment 4:** Were there differences for treatment for stroke between patients with CKD and non-CKD?

**Response 4:** In recent guidelines for the early management of patients with acute ischemic stroke, there were no differences in the acute stage treatment according to the presence of CKD (*Stroke*. 2019;50:e344-e418). Our stroke center also treated acute stroke patients according to the recent stroke management guideline.

**Comment 5:** Because I could not realize the data of CKD G3bA3 in Figure 1, could the author show the data of Figure 1 not only 3D graph but also table?

**Response 5:** Thank you for the reviewer’s suggestion. I changed Figure 1 as 3D graph with all specific values.

**Reviewer #4**

The manuscript entitled of “Blood pressure variability (BPV) and early neurological deterioration (END) according to renal function in minor ischemic stroke patients” used a single center, retrospective data to analysis the effect of renal impairment on blood pressure and on early neurological deterioration in minor ischemic stroke cases. The authors found that there is a significant association of BPV to EDV in renal impairment group but not in the normal renal function group. However, several issues need to be addressed before further consideration.

**Comment 1:** The author provided “CKD” related references in the Introduction part and cited the KDIGO guideline to classified the study subjects into 4 “risk groups” (low risk, moderate risk, high risk, and very high risk) in the method part. The low risk group is depicted as “normal renal function” in the method part. In the result part, the author used the term of “impair renal function” to include the moderate, high, and very high risk groups. And all these terms were used in the discussion portion.

My concern is that this complicating terminology may confuse the readers. For example, a subject with eGFR more than 60 ml/min/m2 without albuminuria is not a CKD case by the KDIGO definition. The subjects of “low risk” or “normal renal function” in this study maybe are non-CKD cases.

For CKD cases, the disease severity are usually classified into CKD stage I to V according to eGFR. However, in this study, the author used the risk predicting grouping. To be noticed, the KDIGO guideline do provide evidence-based risk grouping but the classification varies regarding different outcomes. Therefore, I will suggest the method part should include clear definition of the above terminology, criteria for risk grouping, and the data for grouping should consider to be provided as a supplementary table.

**Response 1:** We appreciate the reviewer’s instructive suggestions. According to the reviewer’s comment, we added the clear definition of terminology in this study, criteria for risk grouping. Also, we changed Figure 1 as 3D graph and color table with data of patients included in each group.

“The risk of adverse outcomes is classified in four groups: low, moderate, high, and very high risk group. Low-risk group is defined as eGFR ≥60 ml/min/1.73m^2^ with normoalbuminuria, moderate-risk group as 1) eGFR ≥60 ml/min/1.73m^2^ with microalbuminuria or 2) 45−59 ml/min/1.73m^2^ of eGFR with normoalbuminuria, High-risk group is defined as 1) 30−44 ml/min/1.73m^2^ with normoalbuminuria, 2) 45−59 ml/min/1.73m^2^ with microalbuminruia, or 3) eGFR ≥60 ml/min/1.73m^2^ with macroalbuminuria. Finally, very-high-risk group is defined as 1) eGFR <30 ml/min/1.73m2 with normoalbuminuria, 2) eGFR <45 ml/min/1.73m2 with microalbuminuria, or 3) eGFR <60 ml/min/1.73m2 with macroalbuminuria. Moreover, the low-risk group is recognized as having normal renal function in KDIGO classification. Therefore, we divided study population into the two groups: normal renal function group (low-risk group) and impaired renal function group (moderate, high, and very-high-risk group).”

**Comment 2:** Is the “stroke mechanism” at line 144, 173, of the Result part equals to the parameter of “subtypes of stroke”? I will suggest more deliberation or careful statement regarding the issue.

**Response 2:** Thank you for the reviewer’s meticulous comment. We changed “stroke mechanism” as “stroke subtypes”.

“However, the stroke subtypes did not differ according to KDIGO classification.”

“No differences existed for risk factors or stroke subtypes between those with and without END.”

**Comment 3:** It is confusing that the author used both “SBP SD, SBPV SD", “SBP Cov, SBPV CoV” to name a few in tables, and in different sentences of results and discussion. Do they mean the same data? Or they were generated by different calculating algorithm? Please ensure the methodology and use consistent term. In addition, the BP variation is the key issue of this article. We could not tell if the BPV come from all BP records from every subjects of a group, or each individual’s BPV was in consideration in the analysis. Please include the rationale and citing the calculation method in the Method part.

**Response 3:** We appreciate the reviewer’s comments. SBP SD and SBPV SD mean the same data, and there are no differences in calculating algorithm. Therefore, we corrected SBPV or DBPV as SBP or DBP for the use of consistent term.

Moreover, BPV in this article came from each individual’s BP record. For instance, SBP SD in END group (Table 2) means the average of each subject’s SBP SD. According to the reviewer’s suggestion, we added the calculation method and the reference.

“We calculated the systolic blood pressure (SBP) variability and diastolic blood pressure (DBP) variability, presenting them as standard deviations (SDs) and coefficient of variation (CoV; equal to [SD × 100]/mean). [11] We calculated BPV of each subject, and then calculated the mean of the variability according to the group.”

**Reviewer #5**

In this single-center retrospective cohort study, the authors investigated whether the relationship between END after minor stoke and parameters of BPV was different between with and without impaired kidney function. Several BPV parameters were significantly associated with END in the subgroup with moderate to very high-risk CKD prognosis, but not in the subgroup with low risk (non-CKD) according to KDIGO 2012 classification.

**Comment 1:** The authors described that they evaluated parameters of BPV using the recorded blood pressure (BP) data during the first 5 days after admission. However, END was determined according to the NIHSS score change during 3 days after admission and BPV parameters in the END group were evaluated ignoring BP data after the diagnosis of END. Therefore, the duration obtaining BP data to evaluate BPV would be different between END and non-END groups. I think the period during which BP data were obtained to evaluate BPV parameters should be standardized to the first 3 days in both END and non-END patients.

**Response 1:** We greatly appreciate the reviewer’s pertinent comment. We understand that the reviewer is concerned. First of all, the definition of END within 3 days in our study referred to the definition of previous studies (*Arch Neurol.* 2005;62(3): 393-397, *Clinical Neurology and Neurosurgery* 195 (2020) 105892). Also, we analyzed 5 days of BP measurement, which is thought to be the period of acute stroke stage. Although we defined END as the worsening of NIHSS score within 3 days, it is quite variable between the patients when END occurred. Moreover, since there may be an intervention by the physicians which may influence BP, BP measurement after END might be less reliable. Consequently, we figured that it is reasonable to analyze BP measurement individually before END occurred. Therefore, it might be less advantage to reduce the period of BP measurement to 3 days. Furthermore, our purpose of this investigation was to evaluate the association between BPV and END according to the renal function. Thus, the period of BP measurement would not be affected by renal function.

**Comment 2:** In Table 3, results of multivariate logistic regression analyses in the normal and impaired renal function subgroups were shown. Please show the results of comparison of clinical parameters same as shown in Table 2 in the impaired renal function subgroup. In this subgroup, were there any clinical factors with significant difference except for age, NIHSS, and BPV parameters between patients with and without END? If there are factors which are significantly different between patients with and without END, they should be included in the multivariate logistic regression analysis.

**Response 2:** Thank you for the reviewer’s valuable comment. The results of comparison of clinical variables in the normal renal function group and impaired renal function group are presented as below.

| Variables | Normal renal function  (N=154) | | P value | Impaired renal function  (N=136) | | P value |
| --- | --- | --- | --- | --- | --- | --- |
|  | Non-END  (N=137) | END  (N=17) |  | Non-END  (N=94) | END  (N=42) |  |
| Age | 62.6±12.7 | 67.1±13.1 | 0.175 | 70.9±11.4 | 72.5±10.5 | 0.413 |
| Male sex | 89 (65.0) | 9 (52.9) | 0.481 | 54 (57.4) | 31 (73.8) | 0.103 |
| Hypertension | 85 (62.0) | 13 (76.5) | 0.369 | 76 (80.9) | 33 (78.6) | 0.940 |
| Diabetes mellitus | 27 (19.7) | 4 (23.5) | 0.750 | 40 (42.6) | 17 (40.5) | 0.969 |
| Hyperlipidemia | 60 (43.8) | 10 (58.8) | 0.360 | 60 (63.8) | 16 (38.1) | 0.009 |
| Atrial fibrillation | 14 (10.2) | 2 (11.8) | 0.691 | 20 (21.3) | 7 (16.7) | 0.697 |
| CAD | 14 (10.2) | 2 (11.8) | 0.691 | 18 (19.1) | 10 (23.8) | 0.695 |
| Current smoking | 57 (41.6) | 4 (23.5) | 0.240 | 33 (35.1) | 18 (42.9) | 0.502 |
| Stroke history | 57 (41.6) | 4 (23.5) | 0.771 | 31 (33.0) | 15 (35.7) | 0.908 |
| HbA1c | 5.9±0.9 | 6.1±1.1 | 0.383 | 6.6±1.5 | 6.5±1.4 | 0.672 |
| r-tPA | 4 (2.9) | 1 (5.9) | 0.447 | 4 (4.3) | 3 (7.1) | 0.676 |
| Admission NIHSS | 2 [1−3] | 3 [2−4] | 0.026 | 2 [1−4] | 3 [1−4] | 0.824 |
| Discharge NIHSS | 1 [0−3] | 6 [4−8] | <0.001 | 2 [1−3] | 5 [4−7] | <0.001 |
| TOAST |  |  | 0.545 |  |  | 0.602 |
| LAD | 32 (23.4) | 6 (35.3) |  | 26 (27.7) | 9 (21.4) |  |
| SVO | 53 (38.7) | 8 (47.1) |  | 25 (26.6) | 14 (33.3) |  |
| CE | 23 (16.8) | 1 (5.9) |  | 21 (22.3) | 7 (16.7) |  |
| UD | 21 (15.3) | 1 (5.9) |  | 18 (19.1) | 8 (19.0) |  |
| OD | 8 (5.8) | 1 (5.9) |  | 4 (4.3) | 4 (9.5) |  |
| SBP mean,  mmHg | 135.4±17.6 | 144.8±16.2 | 0.038 | 140.2±18.2 | 146.4±22.8 | 0.091 |
| SBP SD | 11.0±4.8 | 12.6±4.2 | 0.193 | 11.1±4.3 | 14.4±5.0 | <0.001 |
| SBP CoV | 8.2±3.7 | 8.7±2.8 | 0.554 | 7.9±3.0 | 9.9±3.1 | 0.001 |
| DBP mean,  mmHg | 78.9±10.5 | 81.3±11.5 | 0.380 | 77.7±11.6 | 82.8±13.6 | 0.026 |
| DBP SD | 8.6±18.3 | 8.1±3.2 | 0.757 | 7.9±3.0 | 9.0±3.4 | 0.058 |
| DBP CoV | 10.2±13.5 | 9.8±3.1 | 0.770 | 10.4±4.4 | 11.0±4.0 | 0.484 |

In our analysis, all BPV parameters were adjusted for potential factors (*P*<0.20) for END. In normal renal function group, age, admission NIHSS were associated with END. On the other hand, sex, and hyperlipidemia were associated with END in impaired renal function group. The result is as below.

**Table 3.**

| Variables | Normal renal function  (N = 154) | P value | Impaired renal function  (N = 136) | P value |
| --- | --- | --- | --- | --- |
|  | Adjusted OR (95% CI) |  | Adjusted OR (95% CI) |  |
| SBP mean, mmHg | 1.02 (0.99−1.06) | 0.106 | 1.02 (1.00−1.04) | 0.062 |
| SBP SD | 1.07 (0.97−1.19) | 0.188 | 1.21 (1.10−1.33) | <0.001 |
| SBP CoV | 1.05 (0.91−1.22) | 0.479 | 1.27 (1.11−1.46) | <0.001 |
| DBP mean, mmHg | 1.02 (0.98−1.07) | 0.338 | 1.04 (1.00−1.07) | 0.027 |
| DBP SD | 1.00 (0.96−1.03) | 0.813 | 1.15 (1.02−1.30) | 0.026 |
| DBP CoV | 0.99 (0.94−1.05) | 0.791 | 1.05 (0.96−1.15) | 0.299 |

SBP and DBP mean were adjusted for were adjusted for potential factors (P <0.20) for END. In normal renal function group, age and admission NIHSS were adjusted. On the other hand, sex, and hyperlipidemia were adjusted in impaired renal function group.

Moreover, another reviewer suggested that SD and CoV must be adjusted for mean value due to the dependency between mean and SD or CoV. Therefore, SBP and DBP mean were adjusted for age and admission NIHSS in normal renal function group, and adjusted for sex and hyperlipidemia in impaired renal function group. Furthermore, SBP SD, CoV and DBP SD, CoV were additionally adjusted for SBP or DBP mean due to the dependency between mean and SD or CoV.

**Table 3.**

| Variables | Normal renal function  (N = 154) | P value | Impaired renal function  (N = 136) | P value |
| --- | --- | --- | --- | --- |
|  | Adjusted OR (95% CI) |  | Adjusted OR (95% CI) |  |
| SBP mean, mmHg | 1.02 (0.99−1.06) | 0.106 | 1.02 (1.00−1.04) | 0.062 |
| SBP SD* | 1.06 (0.96−1.19) | 0.254 | 1.20 (1.09−1.32) | <0.001 |
| SBP CoV* | 1.08 (0.93−1.25) | 0.298 | 1.30 (1.12−1.50) | <0.001 |
| DBP mean, mmHg | 1.02 (0.98−1.07) | 0.338 | 1.04 (1.00−1.07) | 0.027 |
| DBP SD* | 0.99 (0.94−1.03) | 0.527 | 1.13 (0.99−1.28) | 0.064 |
| DBP CoV* | 0.98 (0.93−1.04) | 0.517 | 1.09 (0.99−1.20) | 0.091 |

SBP and DBP mean were adjusted for were adjusted for potential factors (P <0.20) for END. In normal renal function group, age and admission NIHSS were adjusted. On the other hand, sex, and hyperlipidemia were adjusted in impaired renal function group.

*SBP SD and CoV were additionally adjusted for SBP mean, and DBP SD and CoV were additionally adjusted for DBP mean.

There were some minor changes in odds ratio or P value, but there were no statistically significant changes in this method. It is more reasonable that the adjustment for BPV parameters is performed separately with mean value in SD and CoV. Therefore, we corrected our table as above. We appreciate the reviewer’s instructive comment.

**Comment 3:** Table S1 showed the results of multivariate logistic regression analysis and the authors argued that moderate and very high risk groups in the KDIGO classification were independently associated with END. Please discuss the possible reasons why only moderate and very high-risk group, but not high-risk group, were associated with END.

**Response 3:** We appreciate the reviewer’s instructive comment. As the reviewer’s mentioned, there were some discrepancies in patients with high risk group of CKD patients (30.9% in moderate risk group vs. 18.2% in high risk group vs. 47.1% in very high risk group). It seems to be that the number of high, and very high risk group was small compared with low or moderate risk group. Small sample size in high and very high risk group was a limitation of this study. But in this pilot study, we showed the effects of BPV on END are associated with renal function in minor ischemic stroke patients. To overcome small sample size in high and very high risk group, we divided patients into normal renal function vs. impaired renal function group. Moreover, BPV was associated with END in patients with impaired renal function, but less in those with normal renal function. Although we showed the effect of CKD and BPV on END, there were some discrepancies in patients with high risk group of CKD patients, and it may be caused by small number of high and very high risk group. Further research with large study population must be needed. Therefore, we emphasized the first limitation as below.

“First, because we performed the study at a single center with a small sample size, it is difficult to generalize the results. Especially, the number of high risk and very high risk group was too small. Therefore, the findings require further verification in a larger, prospective study.”

**Comment 4:** Figure 1 showed the distribution of percentage of END in each category of KDIGO classification. Very-high risk group looked like to have high prevalence of END, however, because the number of patients seemed to be quite different between categories and there were only 17 patients in the very-high risk group, Figure 1 might be misleading for the readers. Not only percentage but also absolute number of patients in each KDIGO risk category should be shown.

**Response 4:** We appreciate your careful comment. We changed Figure 1 as 3D graph with detailed number of patients in each KDIGO risk category.

**Comment 5:** The described definition of END described in the Methods section seemed to be slightly different from that in the abstract. Which definition was true in your study?

**Response 5:** The definition of END in this study was an increase at least 1 point in motor power or a total NIHSS score deterioration of ≥2 points. We corrected the definition of END in the Abstract. We apologize for our mistake which were the reviewer’s concerns.

“We enrolled ischemic stroke patients with a National Institutes of Health Stroke Scale of ≤5. END was defined as worsening of ≥1 point in motor power or ≥2 points in total score.”

**Comment 6:** Table 1 showed patient characteristics in this study. Type of medication for hypertension and use of anti-platelet agents should be also shown.

**Response 6:** We appreciate the reviewer’s instructive suggestions. According to the reviewer’s mention, we additionally investigated the type of anti-hypertensive medication and antithrombotic agent before stroke. In our study, there were no differences in the class of anti-hypertensive drugs or antithrombotic agent. Additional data about medication are as below (Statistical analysis in BP lowering agent was performed separately, because there were so many combinations in description of anti-hypertensive agent).

| Characteristic | The risk of KDIGO classification | | | | P value |
| --- | --- | --- | --- | --- | --- |
|  | Low  (N = 154) | Moderate  (N = 97) | High  (N = 22) | Very high  (N = 17) |  |
| BP lowering agent  at admission | 72 (46.8) | 55 (56.7) | 18 (81.8) | 14 (82.4) |  |
| RAS inhibitors | 54 (75.0) | 37 (67.3) | 10 (55.6) | 7 (50.0) | 0.172 |
| β-blockers | 9 (12.5) | 12 (21.8) | 3 (16.7) | 5 (35.7) | 0.175 |
| CCBs | 43 (59.7) | 38 (69.1) | 12 (66.7) | 8 (57.1) | 0.681 |
| Diuretics | 17 (23.6) | 8 (14.5) | 2 (11.1) | 2 (14.3) | 0.448 |
| Antithrombotic agent at admission | 54 (35.1) | 47 (48.5) | 13 (59.1) | 12 (70.6) | 0.801 |
| Antiplatelet agent | 39 (72.2) | 39 (83.0) | 9 (69.2) | 10 (83.4) |  |
| Anticoagulation | 12 (22.2) | 6 (12.8) | 3 (23.1) | 1 (8.3) |  |
| Both | 3 (5.6) | 2 (4.2) | 1 (7.7) | 1 (8.3) |  |

RAS, renin-angiotensin system; CCBs, calcium channel blockers.

**Comment 7:** In the Results and Discussion section, the terms of “SBPV” and “DBPV” were used but they would be SBP and DBP.

**Response 7:** Thank you for the reviewer’s comment. We changed “SBPV” and “DBPV” as “SBP” and DBP” in the Results and Discussion section.

**Reviewer #6**

The article Blood Pressure Variability and Early Neurological Deterioration according to Renal Function in Minor Ischemic Stroke Patients by Ryu J.-C. tries to confirm the interrelationship between kidney function and brain damage. The authors retrospectively analyzed data of patients who were admitted hospital with acute minor ischemic stroke. Although the study's hypothesis sounds promising, the approach to the available data has some significant limitations.

**Comment 1:** Introduction – should be improved. Indeed, kidney failure and brain damage share similar pathophysiological mechanisms that affect microcirculation (you mention some sentences in the discussion), and it is a result of vascular aging and other vascular risk factors that you mention (diabetes, hypertension). Moreover, emerging evidence shows that blood pressure variability is associated with arterial/aortic stiffness (a proxy of vascular age) that alters brain microcirculation and increases permeability in the blood-brain barrier, leading to brain damage.

**Response 1:** We greatly appreciate the reviewer’s comment. We added some sentence in Introduction section about the similarity between kidney and brain, and the effect of BPV for arterial stiffness, brain microcirculation, and change of permeability of BBB.

“Both decreased eGFR and presence of proteinuria are independently associated with the outcomes of stroke. [5,6] In addition to the vascular risk factors including aging, hypertension, and diabetes, the damage to the brain and kidney share similar pathomechanisms that affect microvasculature due to anatomical similarities. [7]”

“On the other hand, blood pressure variability (BPV) is associated with arterial compliance, and affects cerebral microcirculation and blood-brain barrier. [12,13] Previous studies showed that increased BPV in the acute stage of stroke has also been associated with an increased risk for END and poor outcomes of stroke. [14,15]”

**Comment 2:** Methods and Results:

a. The flow chart of the participant inclusion/exclusion would increase readability

**Response 2:** Thank you for reviewer’s comment. We added the flow chart of the study population as Supplementary Figure 1 for the reader’s readability.

“We excluded 15 patients with insufficient medical histories, and 16 patients who were receiving hemodialysis. Thus, we included 290 patients in the final analysis (S1 Fig.).”

**Comment 3:** Even though you acknowledge as a limitation that you had only one measurement of UACR, it is a considerable drawback of the study. Based on this single measurement, you then divide patients into risk groups. Additionally, you don’t mention the anthropometrical measurements of the patients. The thing is that obese individuals seem to have higher creatinine excretion in the urine and, therefore, false low UACR.

**Response 3:** We appreciate the reviewer’s careful comment. As the reviewer’s comment, one of the main limitations is that there were no follow-up data of UACR. Follow-up UACR data might influence the results of our study. Also, we added another limitation about the obesity and false positive UACR.

“Third, it is well known that obesity has been associated with low-grade false positive albuminuria. Additional test for the obese patients would have improved our study more clearly. [23]”

**Comment 4:** Blood pressure measurements – was it one measurement every 6 to 8 hours? Usually, standardized blood pressure should be performed, meaning three values in a row should be taken, and the average BP should be calculated.

**Response 4:** Thank you for the reviewer’s comment. As the reviewer’s mentioned, the standard BP measurement for the patients of outpatient department is to be measured three times in a row. However, in real clinical practice, we measured blood pressure every 6 to 8 hours because all patients in this study were admitted to our stroke center (not outpatient department). However, for accurate BP measurement, all BP measurements were performed in a resting, comfortable state with quiet environment. Therefore, we additionally mentioned about BP measurement.

“BP was measured using a validated, calibrated, automatic, and noninvasive BP-monitoring device (IntelliVue MP50; Philips MedizinSysteme, Böblingen, Germany) in the acute stroke unit and general ward according to our local stroke center’s protocol as follows; all BP measurements were performed in a resting, comfortable state with quiet environment; during the patient’s stay in the acute stroke unit, BP was regularly measured every 6 hours;”

**Comment 5:** You have divided patients into groups according to the KDIGO classification and had only 22 patients and 17 patients in high-risk and very high-risk groups, respectively. These numbers are too low to make conclusions about trends among groups. What is noteworthy is that you had 30 (30.9%) patients in the moderate CKD group with END, while only 8 (or 47.7%) patients with END were in the very high-risk group. I suppose you could try to analyze eGFR as a continuous variable without classifying it and show the connection between END and BPV.

Response 5: Thank you for the reviewer’s suggestion. As the reviewer’s commented, due to small sample size in high and very-high risk group, cautious interpretation must be needed in thist study. We mentioned about this inevitable limitation in Discussion section.

Moreover, we tried to analyze eGFR as a continuous variable without classifying it and show the association between BPV and END (END ~ Age + admission NIHSS + eGFR * each BPV parameter separately [SBP mean, SBP SD, SBP CoV, SBP ARV, DBP mean, DBP SD, DBP CoV, DBP mean]). In univariable analysis, odds ratio and 95% CI of eGFR for END was 0.98 (0.97-1.00), and p-value was **0.016**. The results of multivariable analysis are as below (Each BPV parameters were adjusted for age, admission NIHSS, and eGFR).

| BPV parameter | Variable | OR (95% CI) | P value | P for Interaction |
| --- | --- | --- | --- | --- |
| SBP mean | eGFR | 0.99 (0.97−1.01) | 0.218 | 0.057 |
|  | SBP mean | 1.02 (1.01−1.04) | 0.009 |  |
| SBP SD | eGFR | 0.98 (0.97−1.00) | 0.100 | 0.592 |
|  | SBP SD | 1.13 (1.06−1.20) | <0.001 |  |
| SBP CoV | eGFR | 0.99 (0.97−1.00) | 0.125 | 0.195 |
|  | SBP CoV | 1.14 (1.04−1.24) | 0.003 |  |
| DBP mean | eGFR | 0.99 (0.97−1.01) | 0.240 | 0.060 |
|  | DBP mean | 1.04 (1.01−1.07) | 0.005 |  |
| DBP SD | eGFR | 0.99 (0.97−1.01) | 0.224 | 0.026 |
|  | DBP SD | 1.00 (0.98−1.02) | 0.890 |  |
| DBP CoV | eGFR | 0.99 (0.97−1.01) | 0.223 | 0.039 |
|  | DBP CoV | 1.00 (0.97−1.03) | 0.989 |  |

The purpose of this study was that the investigation about the association between BPV and END according to the renal function. When we defined renal function as continuous variable (eGFR value), there were some interactions between eGFR and BPV parameters. Moreover, eGFR had a possible trend for the occurrence of END.

It is well-known that eGFR and albuminuria are independently associated with the stroke outcomes. However, we aimed to investigate the effect of eGFR and albuminuria since these two parameters are complementary for predicting renal outcome and both well predict the progression to end-stage renal disease. Moreover, categorial variables (including eGFR and albuminuria both) are easier to apply for the clinical practice. Therefore, we analyzed using the KDIGO classification for the prediction of END despite of the small number in risky patients.

**Comment 6:** Prognostic factors of END – the main limitation of this analysis is again too small sample numbers in high and very high-risk groups to conclude.

**Response 6:** We sincerely appreciate the reviewer’s comment. As the reviewer mentioned, small sample size in high and very high risk group was indeed a limitation of this study that we have mentioned in the limitation section of the manuscript. But in this pilot study, we showed the effects of BPV on END are associated with renal function in minor ischemic stroke patients. Due to small sample size in high and very high risk group, we divided patients into normal renal function vs. impaired renal function group. Moreover, BPV was associated with END in patients with impaired renal function, but less in those with normal renal function. Although we showed the effect of CKD and BPV on END, it is also true that the number of high and very high risk group was small. Therefore, we emphasized the first limitation as below.

“First, because we performed the study at a single center with a small sample size, it is difficult to generalize the results. Especially, the number of high risk and very high risk group was too small.”

**Comment 7:** Table 3, in logistic regression, the BPV should be adjusted for diabetes even though diabetes was not significant in univariate analysis. Diabetes causes autonomic dysfunction that per se affects blood pressure variability. Hypertension and sex should also be included as covariates in logistic regression.

**Response 7:** We agree the reviewer’s suggestion, and we additionally adjusted for sex, hypertension, and diabetes. There were some minor changes in odds ratio and P value, but there were no statistically significant changes. The results are as below.

| Variables | Normal renal function  (N = 154) | P value | Impaired renal function  (N = 136) | P value |
| --- | --- | --- | --- | --- |
|  | Adjusted OR (95% CI) |  | Adjusted OR (95% CI) |  |
| SBP mean, mmHg | 1.03 (0.99−1.06) | 0.101 | 1.02 (1.00−1.04) | 0.050 |
| SBP SD | 1.05 (0.95−1.16) | 0.345 | 1.21 (1.10−1.34) | <0.001 |
| SBP CoV | 1.03 (0.90−1.18) | 0.690 | 1.28 (1.11−1.47) | <0.001 |
| DBP mean, mmHg | 1.04 (0.99−1.09) | 0.165 | 1.05 (1.01−1.09) | 0.013 |
| DBP SD | 1.00 (0.96−1.04) | 0.980 | 1.15 (1.01−1.29) | 0.029 |
| DBP CoV | 1.00 (0.94−1.06) | 0.966 | 1.04 (0.95−1.14) | 0.352 |

All BPV parameters were adjusted for age, sex, hypertension, diabetes, and admission NIHSS.

On the other hand, other reviewer also mentioned about the adjustment for confounding factors, but suggested methods were different. About this important matter, we consulted a statistician, and the reply was that when subgroup analysis is performed, BPV parameters must be separately adjusted for each confounding factors according to the group. Therefore, we separately analyzed the univariable analysis in normal renal function group and impaired renal function group. Age and admission NIHSS were the potential factors (P<0.20) in normal renal function group. On the other hand, sex and hyperlipidemia were the potential factors in impaired renal function group. Therefore, SBP and DBP mean were adjusted for age and admission NIHSS in normal renal function group, and adjusted for sex and hyperlipidemia in impaired renal function group.

Also, other different reviewer suggested that SD and CoV must be adjusted for mean value because of dependency between mean value and SD or CoV. Furthermore, SBP SD, CoV and DBP SD, CoV were additionally adjusted for SBP or DBP mean due to the dependency between mean and SD or CoV.

**Table 3.**

| Variables | Normal renal function  (N = 154) | P value | Impaired renal function  (N = 136) | P value |
| --- | --- | --- | --- | --- |
|  | Adjusted OR (95% CI) |  | Adjusted OR (95% CI) |  |
| SBP mean, mmHg | 1.02 (0.99−1.06) | 0.106 | 1.02 (1.00−1.04) | 0.062 |
| SBP SD* | 1.06 (0.96−1.19) | 0.254 | 1.20 (1.09−1.32) | <0.001 |
| SBP CoV* | 1.08 (0.93−1.25) | 0.298 | 1.30 (1.12−1.50) | <0.001 |
| DBP mean, mmHg | 1.02 (0.98−1.07) | 0.338 | 1.04 (1.00−1.07) | 0.027 |
| DBP SD* | 0.99 (0.94−1.03) | 0.527 | 1.13 (0.99−1.28) | 0.064 |
| DBP CoV* | 0.98 (0.93−1.04) | 0.517 | 1.09 (0.99−1.20) | 0.091 |

SBP and DBP mean were adjusted for were adjusted for potential factors (P <0.20) for END. In normal renal function group, age and admission NIHSS were adjusted. On the other hand, sex, and hyperlipidemia were adjusted in impaired renal function group.

*SBP SD and CoV were additionally adjusted for SBP mean, and DBP SD and CoV were additionally adjusted for DBP mean.

This result also showed no statistically significant differences with minor change of odds ratio and P value. Therefore, according to the statistician’s reply, we chose this model that BPV parameters are separately adjusted for each confounding factor in each group. We sincerely appreciate the reviewer’s instructive and careful comment.

**Comment 8:** Discussion – you claim that “among those patients classified as very high risk, near half showed END.” It sounds like a solid and vital statement. However, you refer only to 8 patients from 17 in the very high-risk group. I suppose that you should tone down this statement.

**Response 8:** Indeed, the number of very high risk group is small. As the reviewer suggested, “among those patients classified as very high risk, near half showed END” seems to be solid and vital statement. Therefore, we change this sentence as below.

“Among those patients classified as very high risk in our study, a considerable proportion of patients showed END.”
